# Blood gas parameters during cardio-pulmonary resuscitation in a post hoc analysis-porcine study with data pooled from two studies: increased acidosis with repeated adrenaline injections compared to a control group during prolonged advanced life support

**DOI:** 10.1016/j.resplu.2026.101459

**Published:** 2026-08-24

**Authors:** Felicia Feurstein, Adam Åkerman, Jakob Lundager Forberg, Jan Belohlavek, Mikuláš Mlček, Carl-David Dolata, Henrik Wagner

**Affiliations:** a Department of Development, Halmstad Hospital, Sweden; b Department of Cardiology, Helsingborg Hospital, Sweden; c Department of Emergency Medicine, Helsingborg Hospital, Sweden; d Department of Physiology, 1st Faculty of Medicine, Charles University, Albertov 5, Prague 2 128 00, Czech Republic; e The Second Department of Medicine – Department of Cardiovascular Medicine, First Faculty of Medicine, Charles University, Prague and General University Hospital, Prague, Czech Republic; f Institute for Heart Diseases, Wroclaw Medical University, Wrocław, Poland; g Department of Cardiology, Skane University Hospital, Lund, Sweden; h Clinical Sciences Medical Faculty, Lund University, 222 42 Lund Sweden

**Keywords:** Cardiac arrest, Cardiopulmonary resuscitation, Adrenaline, Blood gas analysis, Acidosis, Return of spontaneous circulation

## Abstract

**Background:**

Cardiopulmonary resuscitation (CPR) guidelines recommend an injection of 1 mg of adrenaline every 3–5 min in a cardiac arrest situation. Adrenaline increases return of spontaneous circulation (ROSC) and induces a more acidotic state in cardiac arrest situations. Blood gas analysis is a useful tool to assess the metabolic state during CPR.

Most experimental studies have been conducted with a single injection of adrenaline. However, little is known about how blood gas parameters are affected by repeated adrenaline injections during prolonged CPR.

**Methods:**

This was a pooled post-hoc analysis from two studies, in which repeated injections of adrenaline and saline as a control were administered during 16- or 21-min of CPR according to current guidelines. Sixty pigs were induced with cardiac arrest, randomised to either adrenaline or saline. Arterial blood gases were drawn at baseline, after the second and fourth injection of adrenaline/saline, and after 5 and 10 min of ROSC. The analysis included pH, Base excess, Bicarbonate, lactate, partial pressure of carbon dioxide, partial pressure of oxygen, oxygen saturation, haemoglobin, and potassium.

**Results:**

Sixteen/twenty-four animals (66.7%) obtained ROSC in the control group and 17/36(47.2%) in the adrenaline group (ns). Adrenaline resulted in significantly lower pH, base excess, and bicarbonate higher lactate, carbon dioxide, haemoglobin and potassium, compared to a control-group among animals that achieved ROSC, but not in non-ROSC groups.

**Conclusion:**

Repeated adrenaline injections was associated with a more acidotic profile and higher potassium and haemoglobin levels in animals that achieved ROSC during prolonged CPR compared with the control-group. However, this was not seen in the non-ROSC groups.

## Highlights

• Adrenaline injections create a more acidotic environment in lengthy cardiac arrest.
• The most acidotic state was seen in those animals obtaining ROSC receiving adrenaline.
• Adrenaline increase haemoglobin compared to the control group.

## Introduction

Blood gas analysis plays a crucial role during cardiopulmonary resuscitation (CPR). In animal studies, metabolic acidosis, hyperlactatemia,^1^ hyperkalaemia,^2^ decreased bicarbonate (HCO_3_^–^),^3^ and increased haemoglobin (Hb) have been observed during CPR.^4^ According to current advanced life support (ALS) guidelines, a 1 mg dose of adrenaline (ADR) is recommended intravenously or intraosseous every 3–5 min during CPR.^5^

In an experimental study, animals receiving a single injection of ADR during CPR had significantly lower pH and higher lactate levels compared to controls.^6^ In contrast in another study when a single dose of ADR was administered, no difference in arterial pH, venous pH, partial pressure of carbon dioxide (pCO_2_) or lactate was seen compared to the control group. Further initially higher potassium (K^+^) levels were seen in the ADR group.^7^

Significantly higher pH, lower pCO_2_, K^+^ and lactate in patients achieving ROSC compared to non-ROSC patients have been reported during CPR in out-of-hospital cardiac arrest (OHCA) studies.^8^, ^9^ Poor neurological outcome has been associated with higher lactate levels, lower pH and higher K^+^ levels^8^, ^10^, ^11^ as well as higher pCO_2_, lower hydrogen bicarbonate (HCO_3_^–^), and lower base excess (BE).^8^ In most of these studies, blood gas analysis was performed only once, usually within minutes of arrival at the emergency department.

Adrenaline administration, compared with placebo, increases short-term survival^12^, ^13^ and survival to discharge in OHCA, but is also associated with greater neurological impairment.^13^

Despite the negative effects of ADR such as worsen myocardial dysfunction,^14^ impaired sublingual and cerebral microcirculatory blood flow during CPR and after ROSC, in experimental studies,^6^, ^15^ hypoxia in the peripheral tissue due to vasoconstriction causing acid accumulation due to anaerobic metabolism^16^ and its reduced pharmacological effect in an acidotic environment,^17^ administration of ADR during CPR is recommended to improve short-term ROSC.^5^ In a clinical review Ghazala et al. suggested that blood gas analyses cannot be reliably used for predictive clinical decision purposes since there are conflicting data.^18^ Yet another study indicated increased 30-day mortality with pH values ≤6.2 among OHCA-patients obtaining ROSC.^19^ Since most blood gas studies are performed with only one analysis, and different cause of the caridac arrest (CA), the length of ALS-efforts, knowledge about how various blood gas parameters are affected by repeated ADR administrations during prolonged ALS remains limited.

### Aim

This post-hoc pooled analysis aimed to analyse the development of arterial blood gas parameters during prolonged standardised ALS in animals randomised to receive either guideline-based repeated intravenous injections of ADR or saline (NaCl) as a control, and to compare these parameters between animals that achieved ROSC and those that did not, both between the two groups and within each group.

## Material and methods

### Ethics

The first study was approved by Malmö/ Lund Animal Ethics Committee, Sweden (M192-10) and conformed to the Guide for the Care and Use of Laboratory Animals, US National Institute of Health (NIH Publication No. 85-23, revised 1996) and conducted in the experimental laboratory at Biomedical Centre Wallenberg Laboratory Lund University, Sweden. The second study was approved by Charles University, Czech Republic*,* First Faculty of Medicine Institutional Animal Care and Use Expert Committee. The experiments were conducted in the animal experimental laboratory in accordance with Act No. 246/1992 Coll. on the Protection of Animals Against Cruelty, harmonised with the EU directive on the protection of animals used for scientific purposes.^20^

### Animal experiments

The material comprises two experimental studies. The first study has previously been described.^21^, ^22^ In this study, 36 Swedish-bred, pathogen-free, Landrace pigs of both sexes were included investigating the impact of ADR on different physiological parameters compared to a control group during ALS. For detailed methodology, see references.^21^, ^22^ The pigs were randomised into three groups: repetitive injections of ADR at 0.02 mg/kg/dose, ADR at 0.03 mg/kg/dose diluted to 10 ml, or 10 ml NaCl as a control group.

Following preparation and baseline period, ventricular fibrillation (VF) was induced confirmed by electrocardiogram and a prompt loss in blood pressure measured with a 6 F pig-tail catheter in the ascending aorta. VF persisted untreated for 1 min before the start of CPR with mechanical chest compressions (LUCAS 2™, Jolife AB, part of Stryker, Lund, Sweden) and manual ventilation. At 4, 7, 10, and 13 min of ALS, an injection of the assigned drug, according to ALS guidelines^23^ was administered through a cannula in the ear vein. Arterial blood gas samples were collected at baseline, 10, and 15 min of ALS, and were analysed using an ABL 827 blood gas analyser (Radiometer Medical ApS, Copenhagen, Denmark). After 15 min of ALS, the first defibrillation attempt was made. If ROSC was not obtained, a fifth dose of ADR was administered, and up to two additional defibrillation attempts were performed if necessary. During the ROSC period, blood gas samples were collected at 5 and 10 min. The animals were euthanised without regaining consciousness after 15 min of ROSC.

The second study has been described previously, for detailed methodology see 24. Twenty-four domestic pigs of both sexes were included. After a 2-min baseline period, VF was induced verified by electrocardiogram and a prompt loss of blood pressure measured with a 6 F pigtail catheter in the aortic root. After 1 min of untreated VF, mechanical chest compressions (LUCAS 2™, Jolife AB, a part of Stryker, Lund, Sweden) and manual ventilation were initiated according to current ALS guidelines.^25^

The animals were randomised into two groups: ADR 1 mg/10 ml or 10 ml NaCl as a control group, administered via an ear vein cannula after 4, 8, 12, 16, and 20 min of ALS. Arterial blood samples were collected at baseline, 11 min, and 19 min of ALS, and analysed using an ABL 827 blood gas analyser (Radiometer Medical ApS, Copenhagen, Denmark).

The first defibrillation attempt was made after 21 min of ALS; if necessary, up to two additional attempts were performed. Blood gas was drawn after 5 and 10 min in animals that achieved ROSC. The animals were euthanised after 60 min without regaining consciousness.

For full description of the preparation of the experiments, see (Supplement full method description).

### Preparation of the materials

**Fig. 1:**
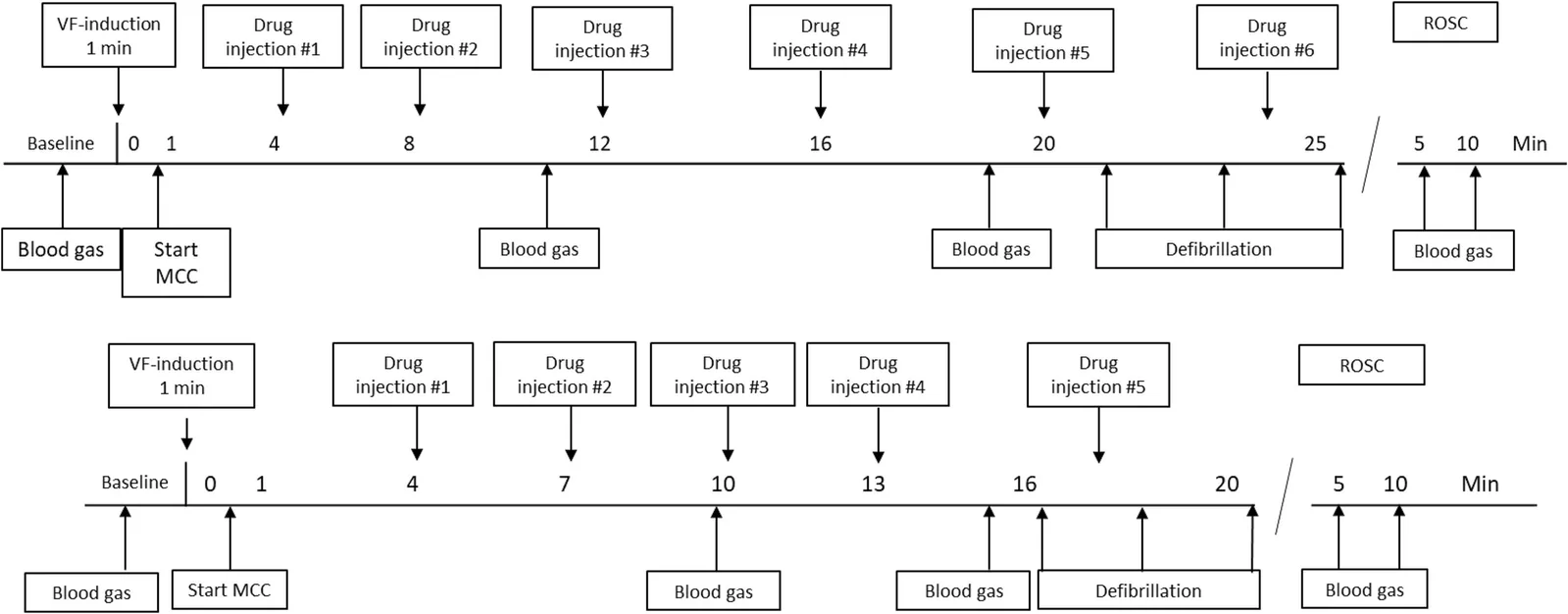
**Timeline of the two studies: administration of adrenaline or saline injections as control. Upper timeline: first study, lower timeline: second study**. Current guidelines were followed, with 4 min of CPR before the first injection. VF = ventricular fibrillation, MCC = mechanical chest compression, ROSC = return of spontaneous circulation, min = minutes.

Included blood gas parameters were pH, BE, HCO_3_^–^, lactate, pCO_2_, partial pressure of oxygen (pO_2_), oxygen saturation (sO_2_), Hb, and K^+^. Due to slightly different timeframes for drug injections and blood gas sampling in the two studies, both timelines are presented (Fig. 1). Blood gas measurements were depicted at baseline, after the second and fourth injection, and at 5- and 10-min post-ROSC.

**Fig. 2:**
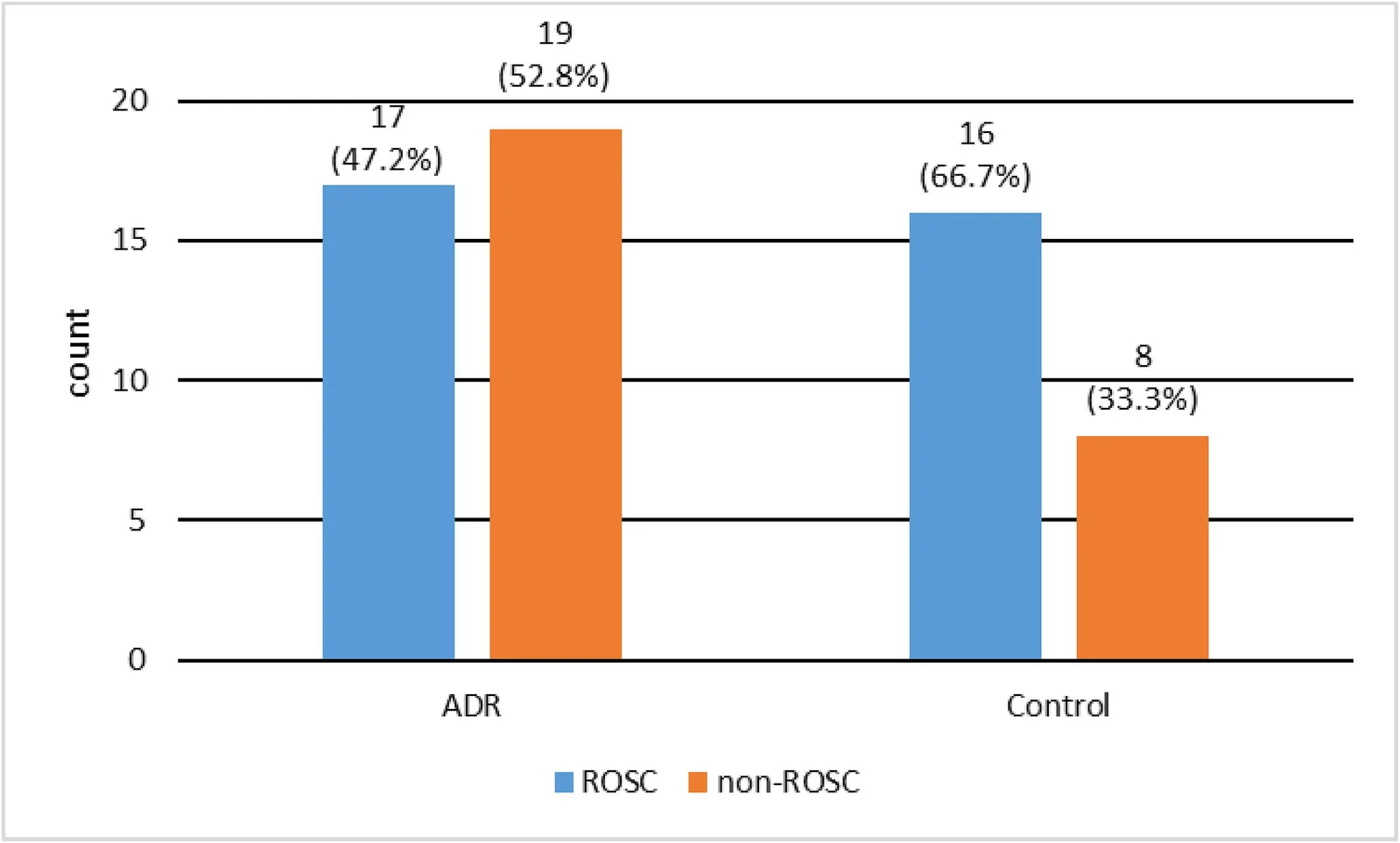
**Distribution of animals in the adrenaline group and control group, showing those that achieved ROSC and non-ROSC**. ADR = Adrenaline, ROSC = return of spontaneous circulation.

The different doses of ADR were combined into one group, hereafter referred to as ADR.

Comparisons regarding animal weight and ROSC versus non-ROSC are shown in Table 1.

**Table 1:** Animal weight between groups randomised to control or ADR and between animals achieving ROSC and non-ROSC.

**All animals**				**ROSC**			**Non-ROSC**		
**Randomisation group (*n*)**	**Control all**^24^	**ADR all**^36^	***P*-value**	**Control**^16^	**ADR**^17^	***P*-value**	**Control**^8^	**ADR**^19^	***P*-value**
Animal weight (kg) (mean ± 1SD)	45.6 ± 8	42.6 ± 8.1	0.162	43.6 ± 8.0	41.1 ± 8.1	0.359	50.1 ± 6.2	44.2 ± 8.8	0.098

**All animals**				**Control group**			**ADR group**		

**CPR outcome (*n*)**	**ROSC**^33^	**Non-ROSC**^27^	***P*-value**	**ROSC**^16^	**Non-ROSC**^8^	***P*-value**	**ROSC**^17^	**Non-ROSC**^19^	***P*-value**

Animal weight (kg) (mean ± 1SD)	42.3 ± 7.5	46.0 ± 8.5	0.082	43.6 ± 8.0	50.1 ± 6.2	0.056	41.1 ± 8.1	44.2 ± 8.8	0.256

ROSC = Return of spontaneous circulation, ADR = adrenaline, SD = standard deviation. Values are expressed as (mean ± SD). Significance was set at *P*-value = 0.05.

**Fig. 3:**
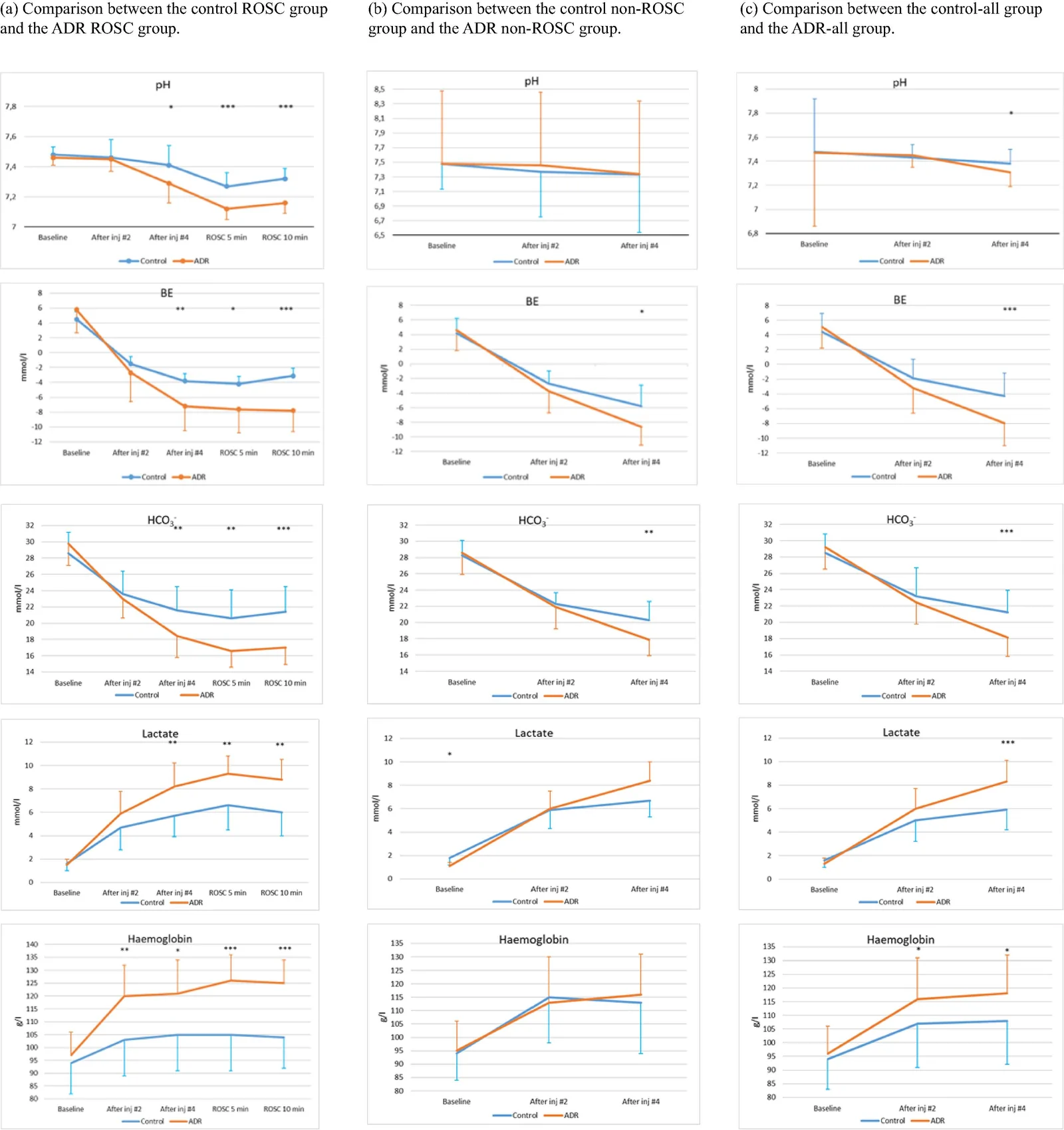

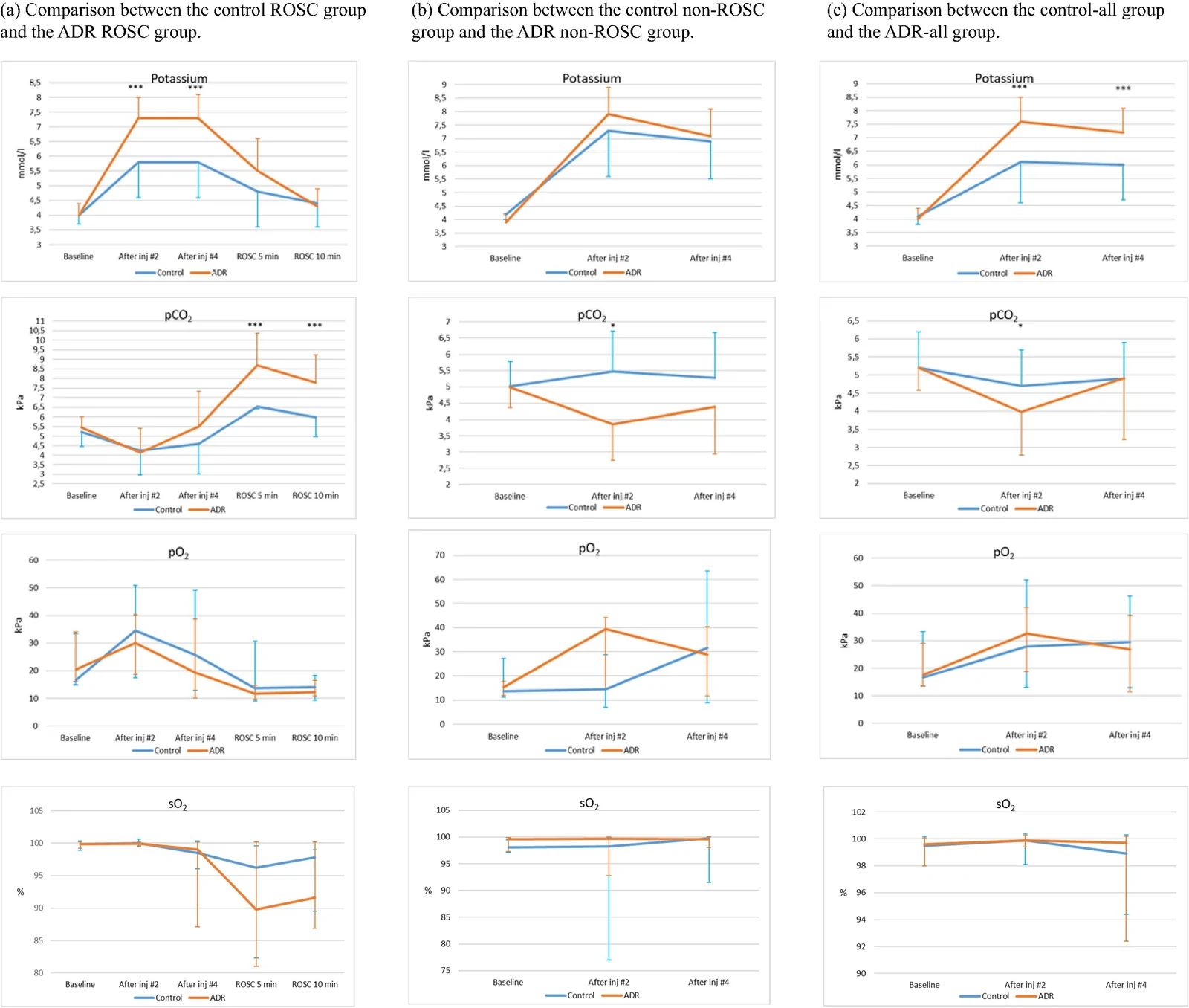
**Differences in, blood gas parameters, haemoglobin and potassium, between the control group and the ADR group**. ROSC = return of spontaneous circulation, BE = base excess, HCO_3_^–^ = bicarbonate, pCO_2_ = partial pressure of carbon dioxide, pO_2_ = partial pressure of oxygen, sO_2_ = saturation of oxygen, kPa = kilo Pascal, pH, BE, HCO_3_^−^, Lactate, haemoglobin, potassium and pCO_2_ are expressed in mean ± 1 SD, pO_2_ and SO_2_ are expressed in median(25–75) quartile. * = *p* < 0.05, ** = *p* < 0.001, *** = *p* < 0.0001.

**Fig. 4:**
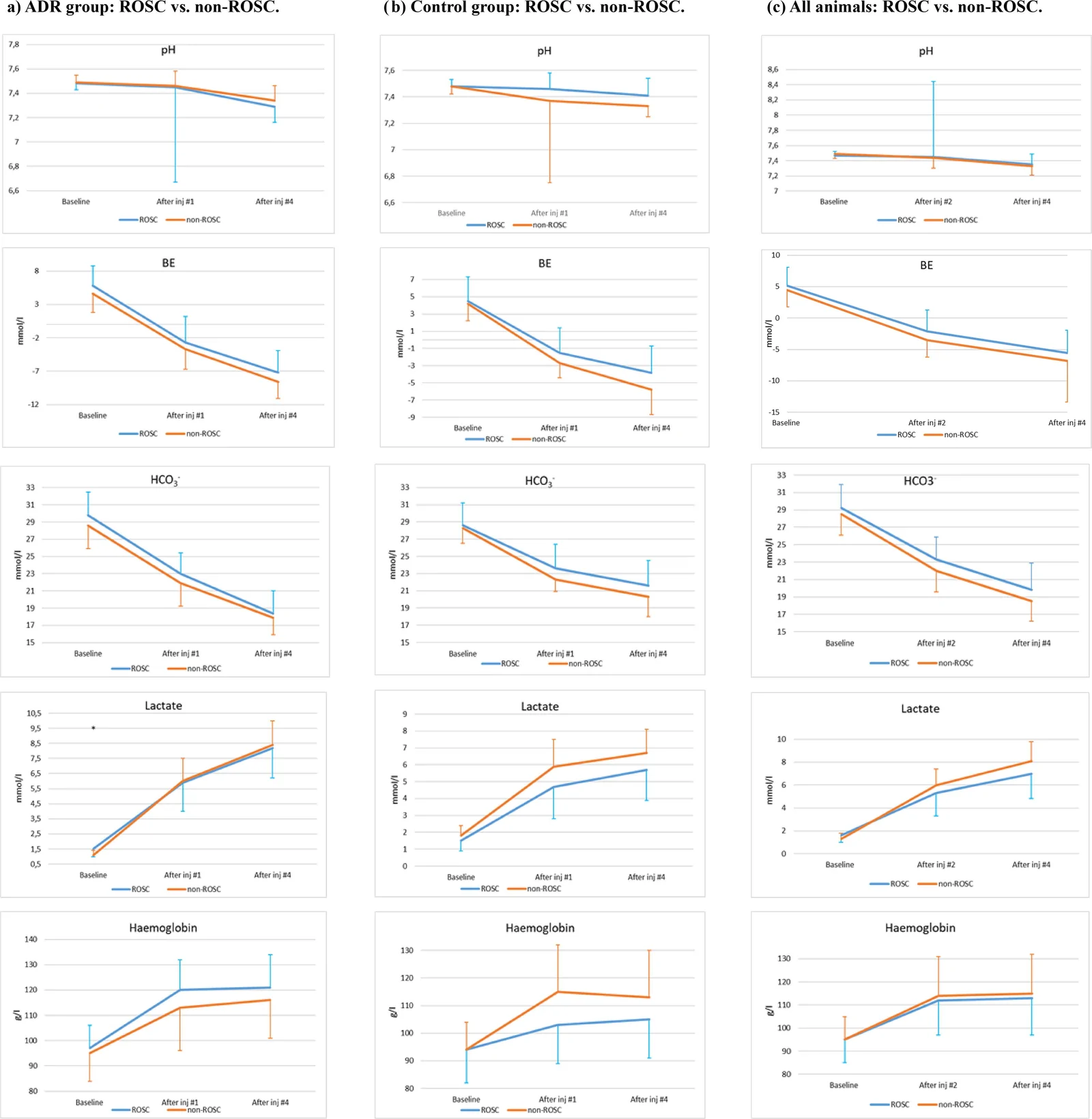

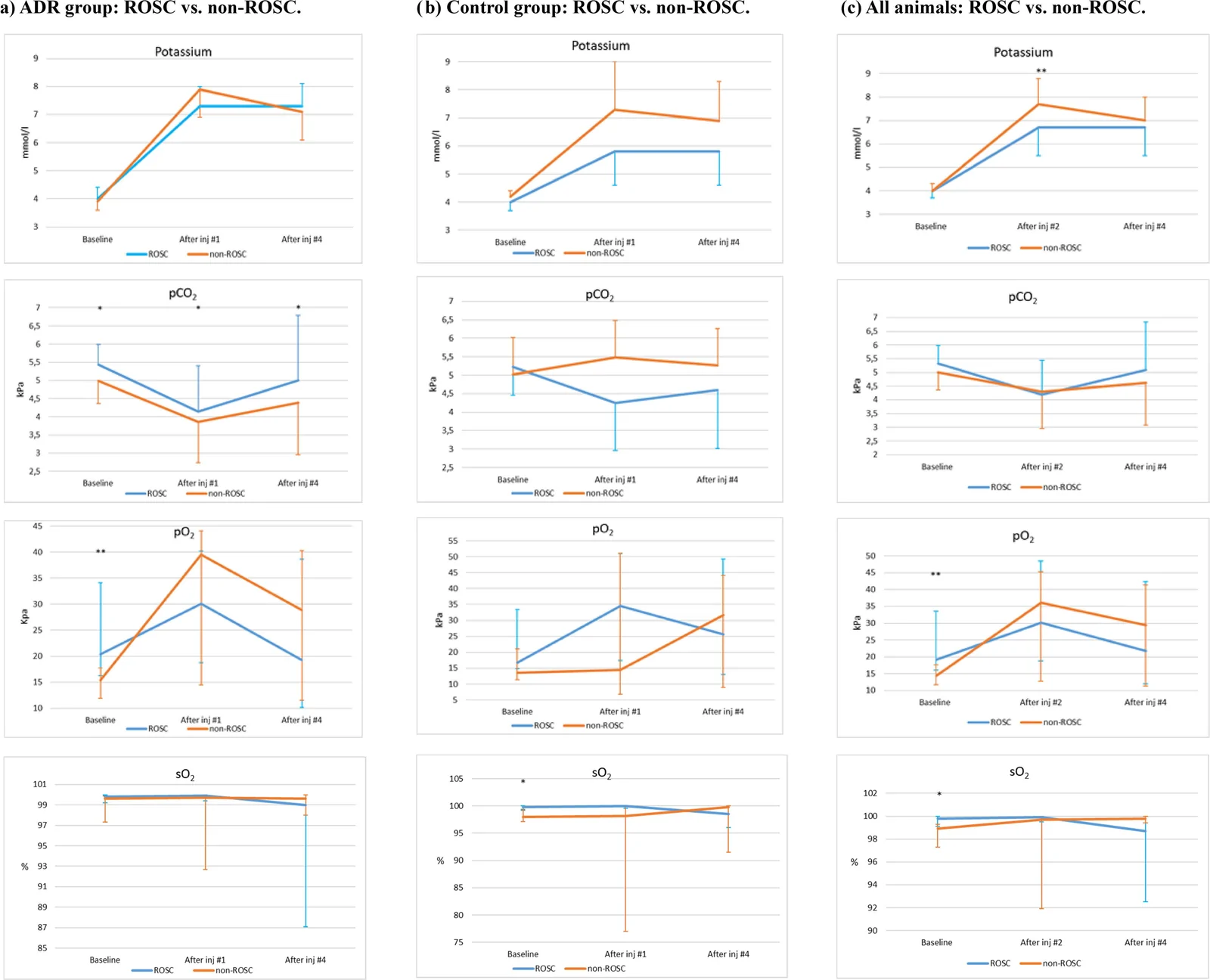
**Comparison of blood gas parameters between ROSC and non-ROSC groups in the ADR- and control group**. ROSC = return of spontaneous circulation, BE = base excess, HCO_3_^–^ = bicarbonate, pCO_2_ = partial pressure of carbon dioxide, pO_2_ = partial pressure of oxygen, sO_2_ = saturation of oxygen, kPa = kilo Pascal, pH, BE, HCO_3_^–^, Lactate, haemoglobin, potassium and pCO_2_ are expressed in mean ± 1 SD, pO_2_ and SO_2_ are expressed in median(25–75) quartile. * = *p* < 0.05, ** = *p* < 0.001.

Blood gas parameters were systematically compared across several groups. Animals treated with ADR were compared with those receiving saline as control. Within each group, animals achieving ROSC were analysed separately from those that did not. Outcomes were compared between all animals achieving ROSC versus all non-ROSC animals, irrespective of treatment group. Finally, direct comparisons were made between ROSC and non-ROSC subgroups within both the ADR and control cohorts. These comparisons are summarised in Tables 2, 4 and Fig. 3, while broader ROSC versus non-ROSC analyses are presented in Tables 3, 5 and Fig. 4.

**Table 2:** Blood gas parameters, haemoglobin and potassium between ROSC, non-ROSC and the combined groups.

	**ROSC (*n* = 33)**			**Non-ROSC (*n* = 27)**			**All (*n* = 60)**		
	**Control (*n* = 16)**	**ADR (*n* = 17)**	***P*-value**	**Control (*n* = 8)**	**ADR (*n* = 19)**	***P*-value**	**Control (*n* = 24)**	**ADR (*n* = 36)**	***P*-value**
**pH (mean ± SD)**									
Baseline	(7.48 ± 0.05)^(16)^	(7.46 ± 0.05) ^(17)^	0.217	(7.48 ± 0.03) ^(7)^	(7.49 ± 0.06) ^(19)^	0.669	(7.48 ± 0.04)^(23)^	(7.47 ± 0.06)^(36)^	0.713
After injection #2	(7.46 ± 0.12)^(16)^	(7.45 ± 0.08)^(17)^	0.757	(7.37 ± 0.06)^(7)^	(7.46 ± 0.12)^(19)^	0.062	(7.43 ± 0.11)^(23)^	(7.45 ± 0.10)^(36)^	0.377
After injection #4	(7.41 ± 0.13)^(16)^	(7.29 ± 0.13)^(17)^	0.012	(7.33 ± 0.08)^(7)^	(7.34 ± 0.12)^(19)^	0.916	(7.38 ± 0.12)^(23)^	(7.31 ± 0.12)^(36)^	0.042
ROSC 5 min	(7.27 ± 0.09)^(16)^	(7.12 ± 0.07)^(17)^	<0.001						
ROSC 10 min	(7.32 ± 0.07)^(15)^	(7.16 ± 0.07)^(17)^	<0.001						

**BE (mmol/L) (mean ± SD)**									
Baseline	(4.5 ± 2.8)^(16)^	(5.8 ± 3.0)^(17)^	0.215	(4.2 ± 2.0)^(7)^	(4.6 ± 2.8)^(19)^	0.726	(4.4 ± 2.5)^(23)^	(5.1 ± 2.9)^(36)^	0.314
After injection #2	(−1.5 ± 2.9)^(16)^	(−2.7 ± 3.9)^(17)^	0.348	(−2.7 ± 1.7)^(7)^	(−3.7 ± 3.0)^(19)^	0.406	(−1.9 ± 2.6)^(23)^	(−3.2 ± 3.4)^(36)^	0.115
After injection #4	(−3.8 ± 3.1)^16)^	(−7.2 ± 3.3)^(17)^	0.004	(−5.8 ± 2.9)^(6)^	(−8.6 ± 2.5)^(19)^	0.031	(−4.3 ± 3.1)^(23)^	(−8.0 ± 3.0)^(36)^	<0.001
ROSC 5 min	(−4.2 ± 4.1)^(16)^	(−7.6 ± 3.2)^(16)^	0.013						
ROSC 10 min	(−3.1 ± 3.3)^(15)^	(−7.8 ± 2.8)^(17)^	<0.001						

**HCO_3_^–^ (mmol/L) (mean ± SD)**									
Baseline	(28.6 ± 2.6)^(15)^	(29.8 ± 2.7)^(16)^	0.237	(28.3 ± 1.8)^(7)^	(28.6 ± 2.7)^(15)^	0.789	(28.5 ± 2.3)^(22)^	(29.2 ± 2.7)^(31)^	0.342
After injection #2	(23.6 ± 2.8)^(14)^	(23.0 ± 2.4)^(15)^	0.571	(22.3 ± 1.4)^(6)^	(21.9 ± 2.7)^(18)^	0.770	(23.2 ± 3.5)^(20)^	(22.4 ± 2.6)^(33)^	0.296
After injection #4	(21.6 ± 2.9)^(13)^	(18.4 ± 2.6)^(17)^	0.003	(20.3 ± 2.3)^(6)^	(17.9 ± 2.0)^(18)^	0.019	(21.2 ± 2.7)^(19)^	(18.1 ± 2.3)^(35)^	<0.001
ROSC 5 min	(20.6 ± 3.5)^(15)^	(16.6 ± 2.0)^(16)^	0.001						
ROSC 10 min	(21.4 ± 3.1)^(13)^	(17.0 ± 2.1)^(17)^	<0.001						

**Lactate (mmol/L) (mean ± SD)**									
Baseline	(1.6 ± 0.6)^(13)^	(1.5 ± 0.5)^(16)^	0.772	(1.8 ± 0.6)^(4)^	(1.1 ± 0.3)^(15)^	0.002	(1.6 ± 0.6)^(17)^	(1.3 ± 0.5)^(31)^	0.057
After injection #2	(4.7 ± 1.9)^(13)^	(5.9 ± 1.9)^(16)^	0.103	(5.9 ± 1.6)^(4)^	(6.0 ± 1.5)^(15)^	0.961	(5.0 ± 1.8)^(17)^	(6.0 ± 1.7)^(31)^	0.068
After injection #4	(5.7 ± 1.8)^(13)^	(8.2 ± 2)^(16)^	0.001	(6.7 ± 1.4)^(4)^	(8.4 ± 1.6)^(15)^	0.072	(5.9 ± 1.7)^(17)^	(8.3 ± 1.8)^(31)^	<0.001
ROSC 5 min	(6.6 ± 2.1)^(11)^	(9.3 ± 1.5)^(16)^	0.001						
ROSC 10 min	(6.0 ± 2.0)^(10)^	(8.8 ± 1.7)^(16)^	0.001						

**Haemoglobin (g/L) (mean ± SD)**									
Baseline	(94 ± 12)^(15)^	(97 ± 9)^(17)^	0.391	(94 ± 10)^(7)^	(95 ± 11)^(19)^	0.813	(94 ± 11)^(23)^	(96 ± 10)^(36)^	0.449
After injection #2	(103 ± 14)^(15)^	(120 ± 12)^(17)^	0.001	(115 ± 17)^(7)^	(113 ± 17)^(19)^	0.794	(107 ± 16)^(23)^	(116 ± 15)^(36)^	0.018
After injection #4	(105 ± 14)^(15)^	(121 ± 13)^(17)^	0.002	(113 ± 19)^(7)^	(116 ± 15)^(19)^	0.697	(108 ± 16)^(23)^	(118 ± 14)^(36)^	0.009
ROSC 5 min	(105 ± 14)^(15)^	(126 ± 10)^(17)^	<0.001						
ROSC 10 min	(104 ± 12)^(15)^	(125 ± 9)^(17)^	<0.001						

**Potassium (mmol/L) (mean ± SD)**									
Baseline	(4.0 ± 0.3)^(13)^	(4.0 ± 0.4)^(17)^	0.960	(4.2 ± 0.2)^(4)^	(3.9 ± 0.3)^(14)^	0.110	(4.1 ± 0.3)^(17)^	(4.0 ± 0.4)^(31)^	0.396
After injection #2	(5.8 ± 1.2)^(13)^	(7.3 ± 0.7)^(17)^	<0.001	(7.3 ± 1.7)^(4)^	(7.9 ± 1.0)^(14)^	0.423	(6.1 ± 1.5)^(17)^	(7.6 ± 0.9)^(31)^	<0.001
After injection #4	(5.8 ± 1.2)^(13)^	(7.3 ± 0.8)^(17)^	<0.001	(6.9 ± 1.4)^(4)^	(7.1 ± 1.0)^(14)^	0.773	(6.0 ± 1.3)^(17)^	(7.2 ± 0.9)^(31)^	<0.001
ROSC 5 min	(4.8 ± 1.2)^(13)^	(5.5 ± 1.1)^(16)^	0.115						
ROSC 10 min	(4.4 ± 0.8)^(12)^	(4.3 ± 0.6)^(16)^	0.834						

**pCO_2_ (kPa) (mean ± SD)**									
Baseline	(5.22 ± 0.77)^(16)^	(5.44 ± 0.55)^(17)^	0.331	(5.02 ± 0.76)^(7)^	(4.99 ± 0.62)^(19)^	0.895	(5.20 ± 0.8)^(23)^	(5.20 ± 0.62)^(36)^	0.996
After injection #2	(4.24 ± 1.28)^(16)^	(4.15 ± 1.26)^(17)^	0.855	(5.48 ± 1.24)^(7)^	(3.86 ± 1.12)^(19)^	0.004	(4.69 ± 1.30)^(23)^	(3.97 ± 1.18)^(36)^	0.042
After injection #4	(4.60 ± 1.58)^(16)^	(5.50 ± 1.80)^(17)^	0.139	(5.27 ± 1.41)^(7)^	(4.39 ± 1.44)^(19)^	0.175	(4.90 ± 1.48)^(23)^	(4.91 ± 1.69)^(36)^	0.981
ROSC 5 min	(6.53 ± 0.13)^(16)^	(8.68 ± 1.68)^(17)^	<0.001						
ROSC 10 min	(5.99 ± 1.02)^(16)^	(7.78 ± 1.45)^(17)^	<0.001						

**pO_2_ (kPa) Median (25–75)q**									
Baseline	16.7(14.9–33.4)^(16)^	20.4(16.3–34.1)^(17)^	0.363	13.6(11.3–21.0)^(7)^	15.4(11.9–17.7)^(19^	0.910	16.6(13.6–33.3)^(23)^	17.6(13.5–29.0)^(36)^	0.889
After injection #2	34.5(17.4–50.9)^(16)^	30.1(18.8–40.2)^(17)^	0.423	14.4(6.9–51.,1)^(7)^	39.5(14.5–44.1)^(19)^	0.497	27.8(13.2–51.0)^(23)^	32.6(18.7–42.2)^(36)^	0.822
After injection #4	25.7(13.0–49.2)^(16)^	19.3(10.2–38.6)^(17)^	0.488	31.7(8.9–44.2)^(7)^	28.9(11.6–40.3)^(19)^	1.000	29.6(12.9–46.3)^(23)^	26.8(11.5–39.2)^(36)^	0.613
ROSC 5 min	13.7(9.1–30.6)^(16)^	11.8(9.6–14.8)^(17)^	0.402						
ROSC 10 min	14.1(9.3–18.3)^(16)^	12.2(11.0–16.6)^(17)^	0.794						

**sO_2_ (%) Median (25–75)q**									
Baseline	99.8(98.9–100.3)^(16)^	99.8(99.2–100.2)^(17)^	0.901	98.0(97.1–99.4)^(7)^	99.6(97.3–99.9)^(19)^	0.572	99.5(98.0–100.2)^(23)^	99.6(98.0–100.1)^(36)^	0.846
After injection #2	100(99.6–100.6)^(16)^	99.9(99.4–100.2)^(17)^	0.382	98.2(77.0–99.9)^(7)^	99.7(92.7–100.2)^(19)^	0.334	99.9(98.1–100.4)^(23)^	99.9(99.4–100.3)^(36)^	0.944
After injection #4	98.5(96.0–100.3)^(15)^	99.0(87.1–100.1)^(17)^	0.628	99.8(91.5–100.0)^(7)^	99.6(98.0–100.1)^(19)^	0.306	98.9(94.9–100.3)^(22)^	99.7(92.4–100.2)^(36)^	0.968
ROSC 5 min	96.2(82.3–99.6)^(16)^	89.8(81.0–96.8)^(17)^	0.127						
ROSC 10 min	97.8(89.5–99.0)^(15)^	91.6(86.9–98.0)^(17)^	0.313						

Values in () indicate the number of samples in each analysis. ROSC = Return of spontaneous circulation, ADR = adrenaline, SD = standard deviation, q = quartile, BE = base excess, HCO_3_^–^ = hydrogen bicarbonate, kPa = kilo Pascal, pCO_2_ = partial pressure of carbon dioxide, pO_2_ = partial pressure of oxygen, sO_2_ = saturation of oxygen. Comparisons were performed using a two-sided *T*-test for pH, BE, HCO_3_^–^, lactate, haemoglobin, potassium, and pCO_2_, and the Mann-Whitney *U* test was used for pO_2_ and sO_2_. Statistical significance was set at 0.05.

**Table 3:** Blood gas parameters between all ROSC and non-ROSC animals, ROSC and non-ROSC within the control group and the ADR group.

	**All**			**Control**			**ADR**		
	**ROSC (*n* = 33)**	**Non-ROSC (*n* = 27)**	***p*-value**	**ROSC (*n* = 16)**	**Non-ROSC (*n* = 8)**	***p*-value**	**ROSC (*n* = 17)**	**Non-ROSC (*n* = 19)**	***p*-value**
**pH (mean ± SD)**									
Baseline	(7.47 ± 0.05)^(33)^	(7.49 ± 0.06)^(26)^	0.218	(7.48 ± 0.05)^(16)^	(7.48 ± 0.03)^(7)^	0.910	(7.48 ± 0.05)^(17)^	(7.49 ± 0.06)^(19)^	0.634
After injection #2	(7.45 ± 0.99)^(33)^	(7.44 ± 0.14)^(26)^	0.602	(7.46 ± 0.12)^(16)^	(7.37 ± 0.62)^(7)^	0.070	(7.45 ± 0.78)^(17)^	(7.46 ± 0.12)^(19)^	0.648
After injection #4	(7.35 ± 0.14)^(33)^	(7.33 ± 0.12)^(26)^	0.667	(7.41 ± 0.13)^(16)^	(7.33 ± 0.08)^(7)^	0.173	(7.29 ± 0.13)^(17)^	(7.34 ± 0.12)^(19)^	0.243

**BE mmol/L (mean ± SD)**									
Baseline	(5.15 ± 2.9)^(33)^	(4.5 ± 2.7)^(26)^	0.372	(4.5 ± 2.8)^(16)^	(4.2 ± 2.0)^(7)^	0.799	(5.8 ± 3.0)^(17)^	(4.6 ± 2.8)^(19)^	0.237
After injection #2	(−2.11 ± 3.4)^(33)^	(−3.5 ± 2.7)^(26)^	0.054	(−1.5 ± 2.9)^(16)^	(−2.7 ± 1.7)^(7)^	0.325	(−2.7 ± 3.9)^(17)^	(−3.7 ± 3.0)^(19)^	0.360
After injection #4	(−5.55 ± 3.6)^(33)^	(−6.79 ± 6.6)^(26)^	0.362	(−3.8 ± 3.1)^(16)^	(−5.8 ± 2.9)^(7)^	0.171	(−7.2 ± 3.3)^(17)^	(−8.6 ± 2.5)^(19)^	0.082

**HCO_3_^–^ mmol/L (mean ± SD)**									
Baseline	(29.2 ± 2.7)^(31)^	(28.5 ± 2.4)^(22)^	0.320	(28.6 ± 2.6)^(15)^	(28.3 ± 1.8)^(7)^	0.761	(29.8 ± 2.7)^(16)^	(28.6 ± 2.7)^(15)^	0.238
After injection #2	(23.3 ± 2.6)^(29)^	(22.0 ± 2.4)^(24)^	0.065	(23.6 ± 2.8)^(14)^	(22.3 ± 1.4)^(6)^	0.283	(23.0 ± 2.4)^(15)^	(21.9 ± 2.7)^(18)^	0.225
After injection #4	(19.8 ± 3.1)^(30)^	(18.5 ± 2.3)^(24)^	0.087	(21.6 ± 2.9)^(13)^	(20.3 ± 2.3)^(6)^	0.348	(18.4 ± 2.6)^(17)^	(17.9 ± 2.0)^(18)^	0.479

**Lactate mmol/L (mean ± SD)**									
Baseline	(1.6 ± 0.6)^(29)^	(1.3 ± 0.5)^(19)^	0.071	(1.6 ± 0.6)^(13)^	(1.8 ± 0.6)^(4)^	0.489	(1.5 ± 0.5)^(16)^	(1.1 ± 0.3)^(15)^	0.009
After injection #2	(5.3 ± 2.0)^(29)^	(6.0 ± 1.4)^(19)^	0.199	(4.7 ± 1.9)^(13)^	(5.9 ± 1.6)^(4)^	0.261	(5.9 ± 1.9)^(16)^	(6.0 ± 1.5)^(15)^	0.923
After injection #4	(7.0 ± 2.2)^(29)^	(8.1 ± 1.7)^(19)^	0.402	(5.7 ± 1.8)^(13)^	(6.7 ± 1.4)^(4)^	0.284	(8.2 ± 2)^(16)^	(8.4 ± 1.6)^(15)^	0.712

**Haemoglobin g/L (mean ± SD)**									
Baseline	(95 ± 10)^(32)^	(95 ± 10)^(26)^	0.821	(94 ± 12)^(15)^	(94 ± 10)^(7)^	0.976	(97 ± 9)^(17)^	(95 ± 11)^(19)^	0.589
After injection #2	(112 ± 15)^(32)^	(114 ± 17)^(26)^	0.636	(103 ± 14)^(15)^	(115 ± 17)^(7)^	0.085	(120 ± 12)^(17)^	(113 ± 17)^(19)^	0.164
After injection #4	(113 ± 16)^(32)^	(115 ± 17)^(26)^	0.722	(105 ± 14)^(15)^	(113 ± 19)^(7)^	0.299	(121 ± 13)^(17)^	(116 ± 15)^(19)^	0.244

**Potassium mmol/L (mean ± SD)**									
Baseline	(4.0 ± 0.3)^(30)^	(4.0 ± 0.3)^(18)^	0.818	(4.0 ± 0.3)^(13)^	(4.2 ± 0.2)^(4)^	0.169	(4.0 ± 0.4)^(17)^	(3.9 ± 0.3)^(14)^	0.510
After injection #2	(6.7 ± 1.2)^(30)^	(7.7 ± 1.1)^(18)^	0.004	(5.8 ± 1.2)^(13)^	(7.3 ± 1.7)^(4)^	0.062	(7.3 ± 0.7)^(17)^	(7.9 ± 1.0)^(14)^	0.082
After injection #4	(6.7 ± 1.2)^(30)^	(7.0 ± 1.0)^(18)^	0.321	(5.8 ± 1.2)^(13)^	(6.9 ± 1.4)^(4)^	0.137	(7.3 ± 0.8)^(17)^	(7.1 ± 1.0)^(14)^	0.449

**pCO_2_ kPa (mean ± SD)**									
Baseline	(5.33 ± 0.66)^(33)^	(5.0 ± 0.64)^(26)^	0.054	(5.22 ± 0.77)^(16)^	(5.02 ± 0.76)^(7)^	0.584	(5.44 ± 0.55)^(17)^	(4.99 ± 0.62)^(19)^	0.026
After injection #2	(4.19 ± 1.25)^(33)^	(4.30 ± 1.35)^(26)^	0.761	(4.24 ± 1.28)^(16)^	(5.48 ± 1.24)^(7)^	0.042	(4.14 ± 1.26)^(17)^	(3.86 ± 1.12)^(19)^	0.467
After injection #4	(5.10 ± 1.73)^(33)^	(4.63 ± 1.56)^(26)^	0.307	(4.60 ± 1.58)^(16)^	(5.27 ± 1.41)^(7)^	0.244	(5.50 ± 1.79)^(17)^	(4.39 ± 1.44)^(19)^	0.048

**pO_2_ kPa (median(25–75))q**									
Baseline	19.2(16.0–33.6)^(33)^	14.4(11.7–17.7^)(26)^	<0.001	16.7(14.9–33.4)^(17)^	13.6(11.3–21.0)^(7)^	0.065	20.4(16.3–34.1)^(17)^	15.4(11.9–17.7)^(19)^	<0.001
After injection #2	30.1(18.8–48.5)^(33)^	36.1(12.8–45.2)^(26)^	0.789	34.5(17.4–50.9)^(17)^	14.4(6.9–51.1)^(7)^	0.308	30.1(18.8–40.2)^(17)^	39.5(14.5–44.1)^(19)^	0.504
After injection #4	21.8(12.0–42.3)^(33)^	29.4(11.3–41.3)^(26)^	0.915	25.7(13.0–49.2)^(17)^	31.7(8.9–44.2)^(7)^	0.820	19.3(10.2–38.6)^(17)^	28.9(11.6–40.3)^(19)^	0.639

**sO_2_% (median(25–75))q**									
Baseline	99.8(99.1–100.2)^(33)^	98.9(97.3–99.9)^(26)^	0.012	99.8(98.9–100.3)^(16)^	98.0(97.1–99.4)^(7)^	0.018	99.8(99.2–100)^(17)^	99.6(97.3–99.9)^(19)^	0.114
After injection #2	99.9(99.5–100.3)^(33)^	99.7(91.9–100.1)^(26)^	0.099	100(99.6–100.6)^(16)^	98.2(77.0–99.9)^(7)^	0.076	99.9(99.4–100)^(17)^	99.7(92.7–100.2)^(19)^	0.510
After injection #4	98.7(92.5–100.3)^(33)^	99.8(99.4–100.2)^(26)^	0.146	98.5(96.0–100.3)^(15)^	99.8(91.5–100.0)^(7)^	0.837	99.0(87.1–100)^(17)^	99.6(98.0–100.1)^(19)^	0.081

Values in () indicate the number of samples in each analysis. ADR = adrenaline, ROSC = return of spontaneous circulation, BE = base excess, HCO_3_^–^ = bicarbonate, pCO_2_ = partial pressure of carbon dioxide, kPa = kilo Pascal, pO_2_ = partial pressure of oxygen, sO_2_ = saturation of oxygen, SD = standard deviation, q = quartile. The *t*-test was used for pH, BE, HCO_3_^–^, lactate, haemoglobin, potassium and pCO_2_. A two-sided Mann-Whitney *U* test was used for pO_2_ and sO_2_. Significance was set at *p* < 0.05.

**Table 4:** Point estimate for blood gas parameters, haemoglobin and potassium between ROSC, non-ROSC and the combined groups.

	**ROSC (*n* = 33)** **ADR compared to control**	**NON-ROSC (*n* = 27)** **ADR compared to control**	**All (*n* = 60)** **ADR compared to control**
**PE (95%CI)**			
**pH**			
Baseline	0.439 (−0.256 to 1.127)	0.191 (−1.058 to 0.679)	0.099 (−0.425 to 0.622)
After injection #2	0.109 (−0.575 to 0.791)	−0.867 (−1.759 to 0.041)	−0.238 (−0.762 to 0.288)
After injection #4	0.924 (0.197–1.638)	0.047 (−0.913 to 0.820)	0.563 (0.020–1.086)
ROSC 5 min	1.980 (1.129–2.810)		
ROSC 10 min	2.367 (1.440–3.270)		

**BE**			
Baseline	−0.441 (−1.129 to 0.253)	−0.157 (−1.023 to 0.712)	−0.271 (−0.796 to 0.255)
After injection #2	0.332 (−0.358 to 1.017)	0.374 (−0.502 to 1.244)	0.428 (−0.103 to 0.955)
After injection #4	1.082 (0.341–1.808)	1.074 (0.095–2.032)	1.205 (0.625–1.776)
ROSC 5 min	0.928 (0.190–1.653)		
ROSC 10 min	1.560 (0.752–2.384)		

**HCO_3_^–^**			
Baseline	−0.434 (−1.144 to 0.282)	−0.124 (−1.021 to 0.775)	−0.267 (−0.815 to 0.283)
After injection #2	0.213 (−0.520 to 0.941)	0.140 (−0.787 to 1.063)	0.299 (−0.261 to 0.856)
After injection #4	1.179 (0.385–1.955)	1.194 (0.194–2.170)	1.261 (0.647–1.865)
ROSC 5 min	1.388 (0.589–2.167)		
ROSC 10 min	1.693 (0.835–2.538)		

**Lactate**			
Baseline	0.109 (−0624 to 0.841)	2.056 (0.736–3.333)	0.593 (−0.018 to 1.197)
After injection #2	0.630 (−1.375 to 0.126)	−0.028 (−1.130 to 1.076)	−0.567 (−1.170 to 0.042)
After injection #4	−1.341 (−2.145 to −0.517)	−1.079 (−2.224 to 0.096)	−1.361 (−2013 to −0.697)
ROSC 5 min	−1.488 (−2.370 to −0.582)		
ROSC 10 min	−1.535 (−2.423 to −0.622)		

**Haemoglobin**			
Baseline	−0.303 (−0.988 to 0.386)	−0.105 (−0.971 to 0.763)	−0.204 (−0.727 to 0.322)
After injection #2	−1.347 (−2.099 to −0.579)	0.117 (−0.752 to 0.983)	−0.650 (−1.184 to −0.111)
After injection #4	−1.174 (−1.908 to −0.424)	−0.174 (−1.041 to 0.695)	−0.726 (−1.263 to −0.180)
ROSC 5 min	−1.758 (−2.558 to −0.938)		
ROSC 10 min	−1.920 (−2.756 to −1.063)		

**Potassium**			
Baseline	−0.019 (−0.741 to 0.704)	0.958 (−0.215 to 2.103)	0.259 (−0.336 to 0.851)
After injection #2	−1.572 (−2.392 to −0.731)	−0.466 (−1.582 to 0.664)	−1.266 (−1.905 to −0.615)
After injection #4	−1.584 (−2.405 to 0.741)	−0.166 (−1.276 to 0.949)	−1.130 (−1.760 to −0.490)
ROSC 5 min	−0.609 (−1.353 to 0.146)		
ROSC 10 min	0.081 (−0.669 to 0.829)		

**pCO_2_**			
Baseline	−0.344 (−1.29 to 0.347)	0.059 (−0.809 to 0.925)	−0.001 (−0.548 to 0.545)
After injection #2	0.064 (−0.619 to 0.747)	1.402 (0.438–2.342)	0.586 (0.022–1.143)
After injection #4	−0.529 (−1.220 to 0.171)	0.619 (−0.271 to 1.496)	−0.007 (−0.553 to 0.540)
ROSC 5 min	−1.489 (−2.256 to −0.704)		
ROSC 10 min	−1.411 (−2.181 to −0.621)		

**pO_2_**			
	*r*	*r*	*r*
Baseline	0.160	−0.028	0.018
After injection #2	−0.144	−0.142	−0.029
After injection #4	−0.125	−0.006	−0.066
ROSC 5 min	−0.151		
ROSC 10 min	−0.047		

**sO_2_**			
Baseline	−0.025	0.119	−0.025
After injection #2	−0.157	0.193	0.009
After injection #4	−0.090	0.210	0.005
ROSC 5 min	−0.270		
ROSC 10 min	−0.184		

ROSC = Return of spontaneous circulation, ADR = adrenaline, PE = Point estimate, CI = confidence interval, BE = base excess, HCO_3_^–^ = hydrogen bicarbonate, pCO_2_ = partial pressure of carbon dioxide, pO_2_ = partial pressure of oxygen, sO_2_ = saturation of oxygen. For normal distributed values, Cohen’s *d* Point estimate was used and for non-normally distributed values *r* was calculated.

**Table 5:** Point estimate for blood gas parameters between all ROSC and non-ROSC animals, ROSC and non-ROSC within the control group and the ADR group.

	**All** **ROSC compared to NON-ROSC**	**Control** **ROSC compared to NON-ROSC**	**ADR** **ROSC compared to NON-ROSC**
**PE (95%CI)**			
**pH**			
Baseline	−0.114 (−0.628 to 0.400)	0.052 (−0.837 to 0.940)	−0.160 (−0.815 to 0.496)
After injection #2	0.141 (−0374 to 0.655)	0.866 (−0.069 to 1.782)	−0.514 (−0.808 to 0.503)
After injection #4	0.083 (−0.431 to −0.597)	0.639 (−0.276 to 1.541)	−0.397 (−1.055 to 0.267)

**BE**			
Baseline	0.236 (−0.281 to 0.751)	0.117 (−0.774 to 1.004)	0.402 (−0.262 to 1.060)
After injection #2	0.429 (0.093–0.947)	0.457 (−0.457 to 1.350)	0.310 (−0.351 to 0.966)
After injection #4	0.241 (−0.276 to 0.756)	0.680 (−0.289 to 1.634)	0.474 (−0.193 to 1.134)

**HCO_3_^–^**			
Baseline	0.280 (−0.271 to 0.827)	0.141 (−0.759 to 1.038)	0.433 (−0.283 to 1.143)
After injection #2	0.520 (−0.032 to 1.068)	0.540 (−0.439 to 1.505)	0.434 (−0.264 to 1.123)
After injection #4	0.477 (−0.069 to 1.020)	0.476 (−0.511 to 1.385)	0.242 (−0.425 to 0.906)

**Lactate**			
Baseline	0.559 (−0.477 to 1.160)	−0.406 (−1.529 to 0.731)	1.005 (0.248–1.748)
After injection #2	−0.394 (−0.989 to 0.206)	−0.668 (−1.803 to 0.488)	−0.035 (−0.739 to 0.670)
After injection #4	−0.544 (−1.144 to 0.061)	−0.635 (−1.768 to 0.518)	−0.134 (−0.838 to 0.572)

**Haemoglobin**			
Baseline	−0.204 (−0.727 to 0.322)	−0.014 (−0.902 to 0.875)	0.182 (−0.475 to 0.837)
After injection #2	−0.650 (−1.184 to −0.111)	−0.820 (−1.733 to 0.111)	0.475 (−0.192 to 1.136)
After injection #4	−0.726 (−1.263 to 0.183)	−0.483 (−1.377 to 0.423)	0.396 (−0.268 to 1.054)

**Potassium**			
Baseline	0.069 (−0.519 to 0.657)	−0.826 (−1.971 to 0.345)	0.241 (−0.471 to 0.949)
After injection #2	−0.898 (−1.510 to −0.277)	−1.151 (−2.327 to 0.058)	−0.649 (−1.371 to 0.093)
After injection #4	−0.301 (−0.891 to 0.292)	−0.899 (−2.050 to 0.280)	0.277 (−0.436 to 0.986)

**pCO_2_**			
Baseline	0.516 (−0.009 to 1.036)	0.252 (−0.643 to 1.140)	0.780 (0.095–1.454)
After injection #2	−0.080 (−0.594 to 0.439)	−0.981 (−1.906 to −0.034)	0.246 (−0.413 to 0.901)
After injection #4	0.270 (−0.247 to 0.785)	−0.438 (−1.331 to 0.465)	0.686 (0.007–1.356)

**pO_2_**			
	*r*	*r*	*r*
Baseline	0.446	−0.390	−0.534
After injection #2	−0.035	−0.223	−0.114
After injection #4	−0.014	−0.056	0.082

**sO_2_**			
Baseline	−0.326	−0.481	−0.267
After injection #2	0.215	−0.370	−0.111
After injection #4	−0.191	−0.045	−0.286

ROSC = Return of spontaneous circulation, ADR = adrenaline, PE = Point estimate, CI = confidence interval, BE = base excess, HCO_3_^–^ = hydrogen bicarbonate, pCO_2_ = partial pressure of carbon dioxide, pO_2_ = partial pressure of oxygen, sO_2_ = saturation of oxygen. For normal distributed values, Cohen’s *d* Point estimate was used and for non-normally distributed values *r* was calculated.

At each measurement point, values were compared within specific subgroups. Comparisons included animals with ROSC in the ADR group versus those with ROSC in the control group (Supplement Table 1); animals without ROSC in the ADR group versus those without ROSC in the control group (Supplement Table 2); and all animals in the ADR group versus all animals in the control group, regardless of outcome (Supplement Table 3).

### Statistics

Categorical data are presented as frequencies and percentages and were analysed using the chi-square test. Variables were tested for normal distribution. Student *t*-test and Mann-Whitney *U* test were used when appropriate. For effect size, Cohen’s *d* point estimate was calculated for normally distributed values and an r-value was calculated for non-normally distributed values. A variance test was used to compare values between the different measurement points in each group. Bonferroni correction was applied for multiple comparisons. Odds Ratio (OR) calculation was used for the ADR and control group comparing ROSC and non-ROSC.

Since slightly different ADR-doses were administered in the first and second study a comparison of the amount of ADR/injection was performed with Student T-test and independent point estimate for comparing ADR-doses between animals in ADR-ROSC and non-ROSC groups.

Statistical significance was set at *p* < 0.05. SPSS version 28 (IBM, New York, NY) was used for statistical analyses, and Excel (Microsoft, Redmond, WA) was used for figures.

## Results

### Baseline characteristics

Twenty-four animals were assigned to the control group and 36 to the ADR group. There was no difference in weight (Table 1) or baseline variables between the groups (Table 2).

In the control group, 16 (66.7%) animals achieved ROSC, compared to 17 (47.2%) in the ADR group OR 2.235(0.765–6.527) 95% CI (*p* = 0.138) (Fig. 2). No statistical difference was seen in ADR between the ROSC group, mean ± 1SD (0.99 ± 0.22) mg/injection and the non-ROSC group mean ± 1SD (0.88 ± 0.14) mg/injection, (*p* = 0.08), PE 0.598(−0.76–1.263) 95%CI.

### Blood gas characteristics: main findings

For all detailed comparisons, see Table 2, Table 3, Table 4, Table 5, Figs. 3, 4, Supplement Tables 1–3, Figs. 2 and 3. Generally, a more acidotic state with lower pH, BE, HCO_3_^–^, higher pCO_2_, Hb and K^+^ was seen in the ADR group compared to the control group where the most pronounced difference was seen between the ROSC-groups.

### Effect size

For effect size see (Tables 4 and 5).

### Variance test

Overall, the change in values between measurement time points was more pronounced in the ADR-ROSC and non-ROSC groups than in the control-ROSC and non-ROSC groups (Supplement Tables 1 and 2). However, this difference was less pronounced in the control-all and ADR-all groups (Supplement Table 3).

## Discussion

In this study, animals undergoing prolonged CPR and randomised to ADR that achieved ROSC exhibited a significantly more acidotic profile, characterised by lower pH, BE, and HCO_3_^–^, along with higher pCO_2_, lactate, Hb and K^+^, compared to the control group that achieved ROSC. None of these metabolic, electrolyte, or Hb differences were observed when comparing animals that achieved ROSC with those that did not within the two randomised groups (ADR vs. control).

### Rosc-rates

Numerically, fewer animals (47.2%) obtained ROSC with ADR injections compared with the control group (66.7%) (ns). This is in contrast to one study, which showed a 90% ROSC rate in the ADR group compared to only 10% in the control group.^26^ Several differences may explain this discrepancy. Pearson et al. used dogs, administered a single dose of ADR (1 mg) after 1 min of CPR, and performed defibrillation after an additional 2 min. In current study, CPR was longer, ADR was given in repeated doses, and no defibrillation attempt was performed after each injection. Human studies in OHCA have shown a significant benefit of ADR in achieving ROSC, short-term survival,^13^, ^27^, ^28^ and hospital discharge, but with more severe neurologic outcomes.^13^ In contrast, early administration of ADR in in-hospital CA, where the time to ADR is generally shorter than in OHCA, was associated with a decreased likelihood of ROSC compared with cases where no ADR was administered within 2 min.^29^

In current study pH values were lower in those obtaining ROSC receiving ADR compared to the control group, furthermore, when comparing ROSC and non-ROSC groups in both control- and ADR group no significant difference was noted. One might expect that there would be a worsening acidotic state in non-ROSC groups, more pronounced in the ADR-group due to the known pro acidotic effect from ADR. The reason for this discrepancy will be speculative but might be due to difference in hemodynamic status. This may indicate that despite a low pH after 15 min of ALS does not necessarily translate into a poor outcome. On the other hand, a newly presented study on OHCA-patients obtaining ROSC with pH ≤ 7.2 indicated an increased 30-day mortality.^19^

In one human study pCO_2_ was an independent predictor of ROSC among OHCA patients.^30^ In current study pCO_2_ was significantly higher in the ADR group in the ROSC period but no other significant differences in pCO_2_ among the other examined groups were evident.

There was no statistical difference in lactate between ROSC and Non-ROSC animals in the ADR- and control group respectively, which is in line with a study from Carden et al. where all animals received ADR.^31^ An OHCA-study concluded that there was no independent association between lactate values during resuscitation efforts and ROSC,^32^ which also may be suggested in current study since no difference was seen between those that obtained ROSC and those that did not. Hence, the acidotic state in current study does not directly determine ROSC.

### Acid-base status

Arterial pH in the ADR-all group at baseline and after the second injection were similar to another study at the same time interval; however, that study administered only a single injection of ADR and evaluated changes only for 10 min.^7^ The acidotic effect was observed not only as decreased pH, BE, and HCO_3_^–^, but also as higher pCO_2_ in animals that obtained ROSC. There was no significant difference in pCO_2_ level when comparing the two groups after the second injection in the current study or in the previous study^7^ at 10 min of CPR. This comparison suggests that the acidosis increases with longer CPR and repeated ADR injections, as has been observed in other experimental CPR studies.^15^, ^33^ Another explanation to the more acidotic state in the ROSC group receiving ADR, may be due to higher blood pressure and perfusion causing a more efficient transportation of metabolites. On the other hand, ADR impairs ETCO_2_ compared to saline,^21^, ^34^ which is known to be a surrogate marker for cardiac output.^35^, ^36^ In another study comparing two different CPR models, BE was lower, however not significant, in the group with higher perfusion pressure which may support this explanation.^37^ Previous studies have suggested that the arterial pH does not accurately reflect the pH value in the tissues during resuscitation.^38^

Lactate levels were significantly higher after the fourth drug injection and at 5 and 10 min of ROSC in the ADR-ROSC group compared to the control group. However, this was not seen in the non-ROSC animals for both groups. Further when comparing lactate values within each group there was a significant increase between each measure point except for the ROSC period which also has been reported in other studies.^1^, ^31^ Ristagno and colleagues showed significantly higher lactate in animals receiving ADR compared to placebo or combined with α- or β-blocking drugs.^6^ This may indicate that ADR has a role in creating a more acidotic environment.

Reduced microcirculation caused by ADRs' vasoconstricting effect further leads to anaerobic metabolism, which, together with the low-flow state during CPR, may in part explain the acidosis. Earlier studies have also shown a decreased effect of ADR during acidosis.^17^ Therefore, repeated ADR injections may reduce the effectiveness of ADR.

Hb levels were statistically significantly higher in animals receiving repeated ADR injections during CPR and ROSC. However, this was not observed in the non-ROSC groups or between ROSC and non-ROSC in both groups. An increase in Hb level during CA and CPR has been noted in earlier studies.^4^, ^39^ The increase in Hb may be attributed to impaired function of the sodium–potassium pump,^4^ vascular endothelial dysfunction caused by reduced circulation, and possibly to elevated endogenous ADR.^40^ The more pronounced increase in the ADR group may be due to a more acidotic environment which was seen in an in vitro experiment from Morin and colleagues.^39^ In human studies, Hb >100 g/L immediately after ROSC was associated with survival and a favourable neurologic outcome.^41^ Current study, however, did not investigate neurologic outcome, and to our knowledge, no other work has specifically analysed Hb changes following repeated ADR injections. The implications of increased Hb during cardiac arrest remain unclear, although Jehle et al. reported a trend toward increased use of ALS interventions to achieve ROSC in animals with greater rises in Hb^4^ which was contrasted by lower mortality and favourable neurologic outcome with higher Hb in clinical cardiac arrest studies.^42^, ^43^, ^44^, ^45^

Higher K^+^ levels were evident in the ADR group compared with the control group after the second and fourth injections. In the study by Lindner et al, which used only a single ADR injection, higher K^+^ levels were observed in the ADR group 4 min after injection; however, subsequent measurements showed no difference, and levels had nearly normalised at 10 min of CPR.^7^ This is consistent with another experimental study, which reported an increase in K^+^ levels 1 min after ADR injection that reverted to baseline after 4 min.^46^ Current study included repeated ADR injections, resulting in a more acidotic state, and likely contributed to the sustained elevation of K⁺ levels in the ADR group throughout the CPR compared with the control group.

Several mechanisms may explain how ADR increases K^+^ levels: acidosis increases extracellular K^+^ by decreasing cellular uptake via reduced activity of the sodium–potassium adenosine triphosphate pump.^16^ This is partly contradicted in another study that suggest that the sodium–potassium adenosine triphosphate pump is hardly affected of pH-values ∼6 to ∼8^47^ while lowest pH-values in current study was around 7.0–7.2; ADR’s effect on pH may further increase K⁺; and α-receptor stimulation promotes K⁺ release from hepatocytes.^48^ One may speculate that individuals who achieve ROSC after ADR injections with elevated K^+^ may be more prone to recurrent CA. Combined with the increased severity of post-myocardial dysfunction,^14^ this raises concern during the initial ROSC period. However, K⁺ levels normalised at 10 min post-ROSC in both groups, making this a transient issue.

### Limitations

This study has some limitations. The study reflects a specific scenario of CA due to VF in a coronary catheterization laboratory setting with a swift response followed by prolonged CPR. Nevertheless, the prolonged CPR and repeated ADR injections used, resemble common CPR situations in humans adhering to current guidelines. There are differences in the two studies in time to administration of ADR/saline, collection of blood gas samples and time to first defibrillation attempt. The blood gas collection differs 1 min after second injection and 4 min after fourth injection, but the different timeframes of the injections of ADR/saline are compliant with current ALS guidelines.^49^ However, the 4 min-difference in longer CPR-time may contribute to a more acidotic state in the third blood gas measurement, yet direct comparisons between the two studies were beyond the scope of this study. There is a difference of 5 min to first defibrillation attempt which may affect ROSC-rate and a difference in blood gas results in the ROSC-period in the two studies, like the 4-min difference for the third blood gas measurement where the time differences may be confounders. Further, this is an animal experimental study with strictly controlled healthy animals, and the results cannot be directly translated to human CA with underlying concomitant diseases. However, the detailed results may be useful from a human perspective, as no such studies have been performed in humans. Furthermore, this study is exploratory with small sample sizes without any statistical power calculation and was not designed to test a specific hypothesis. Finally, the two pooled studies refer to ALS-guidelines from 2010^23^ and 2015.^25^ However, compared to current guidelines 2021^49^ and 2025^50^ there are no differences in aspects of, chest compressions, ventilation, administration of ADR and defibrillation.

## Conclusion

Repeated ADR injections during prolonged CPR were accompanied with a significantly more acidotic state, along with increased Hb and K^+^ levels, compared to the control group. This difference was more pronounced in animals that achieved ROSC, though unclear relevance for ROSC-rates. Conversely, this pattern was not observed when comparing ROSC vs. non-ROSC animals overall or within the control and ADR groups separately.

## CRediT authorship contribution statement

**Felicia Feurstein:** Writing – review & editing, Writing – original draft, Methodology, Formal analysis. **Adam Åkerman:** Writing – review & editing, Formal analysis. **Jakob Lundager Forberg:** Writing – review & editing. **Jan Belohlavek:** Writing – review & editing, Investigation. **Mikuláš Mlček:** Writing – review & editing, Resources, Methodology. **Carl-David Dolata:** Writing – review & editing. **Henrik Wagner:** Writing – review & editing, Methodology, Formal analysis, Conceptualization.

## Generative AI

The manuscript has not been written or edited by generative AI.

## ARRIVE guidelines

The study complies with current ARRIVE guidelines.^51^ For full description of animal preparation see Supplement full method description.

## Consent for publication

All authors have given their consent for publication

## Availability of data and materials

Data are available from the corresponding author upon reasonable request.

## Funding

The study was funded by the Laerdahl Foundation, Thelma Zoegas Foundation (TZ 2012-0104), and Stig and Ragna Gorthon Foundation (2019-2583), as well as by the Cooperatio Intensive Care and Physiology Funding, Charles University, and Institutional support RVO-VFN64165.

## Declaration of competing interest

The authors declare the following financial interests/personal relationships which may be considered as potential competing interests: Henrik Wagner, MD, PhD reports financial support was provided by Laerdal Medical AS, Thelma Zoegas Foundation (TZ 2012-0104), and Stig and Ragna Gorthon Foundation (2019-2583). If there are other authors, they declare that they have no known competing financial interests or personal relationships that could have appeared to influence the work reported in this paper.

## Appendix A Supplementary material

Supplementary Data 1 Full description of the preaparation of the experiments.

Supplementary Data 2 ARRIVE document.

Supplementary Tables Table of variance test.
