# Supplementary material for: Blood gas parameters during cardio-pulmonary resuscitation in a post hoc analysis-porcine study with data pooled from two studies: increased acidosis with repeated adrenaline injections compared to a control group during prolonged advanced life support
Source: Resusc Plus. 2026 Aug 24;31:101459. doi: 10.1016/j.resplu.2026.101459 (PMC13559840; doi:10.1016/j.resplu.2026.101459)
Supplement: Supplementary file 1 [file mmc1.docx]

## *Supplement*

## *Full description of Animal experiments*

The material comprises two experimental studies. The first study has previously been described (1, 2). In this study, 36 Swedish-bred, pathogen-free, Landrace pigs exclusively bred for medical experiments from a dedicated animal farmer were included, investigating the impact of ADR on different physiological parameters compared to a control group during ALS. For detailed methodology and studied parameters, see the references (1, 2). The pigs were randomised into three groups: repetitive injections of ADR at 0.02 mg/kg/dose, ADR at 0.03 mg/kg/dose, or NaCl as a control group. In total 44 animals were used while seven animals were excluded in the ADR and one in the control group. The pigs were fasted overnight with free access to water. At the day of the experiment, they were pre-medicated with Ketaminol 150 mg/10 kg (Ketamine 100 mg/ml, Intervet, Danderyd, Sweden) and Rompun 20 mg/10 kg intramuscularly (Xylazin 20 mg/ml, Bayer AG, Leverkusen, Germany). After induction of anaesthesia with thiopental 12.5 mg/kg/dose (Pentothal 100 mg/ml Abbott, Stockholm, Sweden) the animals were orally intubated with cuffed endotracheal tubes. To maintain anaesthesia, a slow infusion of (Fentanyl 1.25 μg/ml (Fentanyl Pharmalink AB, Stockholm, Sweden) in buffered glucose (25 mg/ml) was started at a rate of 2 ml/min and adjusted as needed. During the anaesthesia, meprobamat (Mebumal DAK Copenhagen Denmark) and/or thiopental was titrated if needed in small bolus doses. The animals were ventilated with a mixture of nitrous oxide (70%) and oxygen (30%) and normal air using a mechanical Siemens-Elema 900B ventilator (Siemens Elema Solna, Sweden) in the volume-controlled mode, adjusted to obtain normoventilation maintaining end tidal carbon dioxide at 4.5 - 5.5 kPa. In addition, the animals were monitored with a three-lead electrocardiogram using an IntelliVue MP90 monitoring system (Philips, Eindhoven, The Netherlands).

Following preparation and baseline period, ventricular fibrillation (VF) was induced using a 9 V direct current (Duracell Battery, Procter & Gamble, Cincinnati OH, USA) between a skin electrode and an intracardiac needle inserted to the epicardium with a stimulation time between 5 – 10 seconds confirmed by electrocardiogram and a prompt loss in blood pressure measured with a 6 F pig-tail catheter in the ascending aorta. VF persisted untreated for 1 minute before the start of CPR with mechanical chest compressions (LUCAS 2™, Jolife AB, part of Stryker, Lund, Sweden) and manual ventilation. At 4, 7, 10, and 13 minutes of ALS, an injection of the assigned drug, according to ALS guidelines (3) was administered through a canula placed in the ear vein. Blood gas samples were collected via an introducer in the femoral artery at baseline and at 5, 10, and 15 minutes of ALS, and were analysed using an ABL 827 blood gas analyser (Radiometer Medical ApS, Copenhagen, Denmark). After 15 minutes of ALS, the first defibrillation attempt was made. If ROSC was not obtained, a fifth dose of ADR was administered, and up to two additional defibrillation attempts were performed if necessary. During the ROSC period, blood gas samples were collected at 5, 10, and 15 minutes. The animals were euthanised without regaining consciousness after 15 minutes of ROSC with 40 mmol potassium injected in a pulmonary artery catheter.

The second study has been described previously, for detailed methodology see (4). Twenty-four domestic pigs exclusively bred for medical experiments from a dedicated animal farmer were included and were fasted overnight with free access to water. After premedication with an intramuscular (i.m.) injection of midazolam 0.3 mg/kg, they were anaesthetised with ketamine (20 mg/kg i.m.). An 18G cannula was inserted in the marginal ear vein, and the pigs were orally intubated with an 8-mm endotracheal tube, connected to a ventilator (Hamilton G5, Hamilton Medical AG, Switzerland) to maintain normoventilation (4.5–5.5 kPa) using a mixture of 75% air and 25% oxygen. Anaesthesia was maintained with continuous intravenous infusions of propofol (6–8 mg/kg/h), midazolam (0.1–0.2 mg/kg/h), and morphine (0.1–0.2 mg/kg/h).

After a 2-minute baseline period, VF was electrically induced using a catheter in the right ventricle, verified by electrocardiogram and a prompt loss of blood pressure measured with a 6 F pigtail catheter in the aortic root. After 1 minute of untreated VF, mechanical chest compressions (LUCAS 2™, Jolife AB, a part of Stryker, Lund, Sweden) and manual ventilation were initiated according to current ALS guidelines (5).

The animals were randomised into two groups: ADR 1 mg or NaCl as a control group, administered via an ear vein canula after 4, 8, 12, 16, and 20 minutes of ALS. Blood samples were collected from an introducer in the femoral artery at baseline, 11 minutes, and 19 minutes of ALS, and analysed using an ABL 827 blood gas analyser (Radiometer Medical ApS, Copenhagen, Denmark).

The first defibrillation attempt was made after 21 minutes of ALS; if necessary, up to two additional attempts were performed. Blood gas was drawn after 5 and 10 minutes in animals that achieved ROSC. The animals were euthanised with 40 mmol potassium after 60 minutes without regaining consciousness.

1.	Hardig BM, Gotberg M, Rundgren M, Gotberg M, Zughaft D, Kopotic R, et al. Physiologic effect of repeated adrenaline (epinephrine) doses during cardiopulmonary resuscitation in the cath lab setting: A randomised porcine study. Resuscitation. 2016;101:77–83.

2.	Wagner H, Gotberg M, Madsen Hardig B, Rundgren M, Carlson J, Gotberg M, et al. Repeated epinephrine doses during prolonged cardiopulmonary resuscitation have limited effects on myocardial blood flow: a randomized porcine study. BMC cardiovascular disorders. 2014;14:199.

3.	Deakin CD, Morrison LJ, Morley PT, Callaway CW, Kerber RE, Kronick SL, et al. Part 8: Advanced life support: 2010 International Consensus on Cardiopulmonary Resuscitation and Emergency Cardiovascular Care Science with Treatment Recommendations. Resuscitation. 2010;81 Suppl 1:e93–e174.

4.	Wagner H, Mlcek M, Krupickova P, Popkova M, Mejstrik A, Boucek T, et al. Adrenaline has a limited effect on myocardial microvascular blood flow: A randomised experimental study in a porcine cardiac arrest model. Resusc Plus. 2025;22:100893.

5.	Soar J, Nolan JP, Bottiger BW, Perkins GD, Lott C, Carli P, et al. European Resuscitation Council Guidelines for Resuscitation 2015: Section 3. Adult advanced life support. Resuscitation. 2015;95:100–47.
