# Supplementary material for: Blood gas parameters during cardio-pulmonary resuscitation in a post hoc analysis-porcine study with data pooled from two studies: increased acidosis with repeated adrenaline injections compared to a control group during prolonged advanced life support
Source: Resusc Plus. 2026 Aug 24;31:101459. doi: 10.1016/j.resplu.2026.101459 (PMC13559840; doi:10.1016/j.resplu.2026.101459)
Supplement: Supplementary file 2 [file mmc2.docx]

The ARRIVE guidelines 2.0: author checklist form for Manuscript Number: RESPLU-D-26-00105

1. **Study design For each experiment, provide brief details of study design including:**
a. The groups being compared, including control groups. If no control group has been used, the rationale should be stated.

**Response:** Stated in Animal experiment section. Animals were randomized either to receive adrenaline or saline as a control group and administered according to current ALS-guidelines during resuscitation.

b. The experimental unit (e.g. a single animal, litter, or cage of animals).

**Response:** Stated in Animal experiment section. Counted animals.

2. **Sample size**
a. Specify the exact number of experimental units allocated to each group, and the total number in each experiment. Also indicate the total number of animals used.

**Response:** Stated in animal experiment section and in supplement full method description. 36 animals were randomized to receive adrenaline and 24 animals to the control group. In total 68 animals were used.

b. Explain how the sample size was decided. Provide details of any a priori sample size calculation, if done.

**Response:** Stated in animal experiment section. No sample size was calculated since this study was a post-hoc pooled analysis from a compound of two studies.

3. **Inclusion and exclusion criteria**
a. Describe any criteria used for including and excluding animals (or experimental units) during the experiment, and data points during the analysis. Specify if these criteria were established a priori. If no criteria were set, state this explicitly.

**Response:** Animals which suffered cardiac arrest before the start of the experiment (induction of ventricular fibrillation), development of severe pulmonary aedema, signs of open foramen ovale were excluded.

b. For each experimental group, report any animals, experimental units or data points not included in the analysis and explain why. If there were no exclusions, state so.

**Response:** In the occasion that values were missing in specific parameters e.g. due to failure in analysing (instrumental fail), this was indicated in the caption section to each table.

c. For each analysis, report the exact value of n in each experimental group

**Response:** This was stated in the animal experimental section. Sixty animals were included in the analysis, where 36 animals were randomized to receive adrenaline and 24 animals randomized to receive salina as a control group.

4. **Randomization**
a. State whether randomisation was used to allocate experimental units to control and treatment groups. If done, provide the method used to generate the randomisation sequence.

**Response:** In the first study the randomization was performed with closed envelopes stating which treatment each animal was allocated to receive. In the second study, it was done by a dice (odd or even numbers).

b. Describe the strategy used to minimise potential confounders such as the order of treatments and measurements, or animal/cage location. If confounders were not controlled, state this explicitly.

**Response:** The assessment was that there were some confounders. This has been described in the limitation section. Regarding the allocation to either adrenaline or saline, when adrenaline was given, an increase in blood pressures was seen.

5. **Blinding**

Describe who was aware of the group allocation at the different stages of the experiment (during the allocation, the conduct of the experiment, the outcome assessment, and the data analysis).

**Response:** The person who performed the randomization was aware of the assigned drug at the start of the experiment.

6. **Outcome measures**
a. Clearly define all outcome measures assessed (e.g. cell death, molecular markers, or behavioural changes).

**Response:** Stated in experiment protocol.

b. For hypothesis-testing studies, specify the primary outcome measure, i.e. the outcome measure that was used to determine the sample size.

**Response:** Not applicable

7. **Statistical methods**
a. Provide details of the statistical methods used for each analysis, including software used.

**Response:** Stated in the statistics paragraph.

b. Describe any methods used to assess whether the data met the assumptions of the statistical approach, and what was done if the assumptions were not met.

**Response:** Since this was a post-hoc pooled explorative analysis no assumptions was done prior analysing the data.

8. **Experimental animals**
a. Provide species-appropriate details of the animals used, including species, strain and substrain, sex, age or developmental stage, and, if relevant, weight.

**Response:** Stated in the Animal experiment paragraph, in result paragraph and table 1. Animals of both sexes were used. There is at least one study which has indicated a lower acidotic state among female animals during cardiac arrest and ALS, compared to male animals (Semenas et al. Acta anesthesiolog Scand 2010;54:343-353). However, in current experiments no such comparison was made.

b. Provide further relevant information on the provenance of animals, health/immune status, genetic modification status, genotype, and any previous procedures.

**Response:** Relevant information regarding provenance has been mentioned in the animal experiment section.

9. **Experimental procedures**

For each experimental group, including controls, describe the procedures in enough detail to allow others to replicate them, including:

a. What was done, how it was done and what was used.

**Response:** Stated in Animal experiments, experimental protocol and in supplemental full description of the animal experiments.

b. When and how often.

**Response:** Stated in Animal experiments, experimental protocol and in supplemental full description of the animal experiments.

c. Where (including details of any acclimatisation periods).

**Response:** Stated in Animal experiments

d. Why (provide rationale for procedures).

**Response:** The rationale for the procedures was to measure the response on ALS-guideline administered adrenaline/saline on specific hemodynamic parameters and blood gas changes during the resuscitation time.

10. **Results**

For each experiment conducted, including independent replications, report:

a. Summary/descriptive statistics for each experimental group, with a measure of variability where applicable (e.g. mean and SD, or median and range).

**Response:** This has been done in the result section, in tables and figures

b. If applicable, the effect size with a confidence interval

**Response:** This has been done in the result section in tables.
