# Supplementary material for: Blood gas parameters during cardio-pulmonary resuscitation in a post hoc analysis-porcine study with data pooled from two studies: increased acidosis with repeated adrenaline injections compared to a control group during prolonged advanced life support
Source: Resusc Plus. 2026 Aug 24;31:101459. doi: 10.1016/j.resplu.2026.101459 (PMC13559840; doi:10.1016/j.resplu.2026.101459)
Supplement: Supplementary file 3 [file mmc3.docx]

**Supplement table 1:** **Ccomparisons of differences in the combined group within the control-ROSC group and the ADR-ROSC group**

|  | | **pH** | | | **BE** | | **HCO_3_^-^** | | **Lactate** | | **pCO_2_** | | **potassium** | | **Haemoglobin** | | **pO_2_** | | **sO_2_** | |
| --- | --- | --- | --- | --- | --- | --- | --- | --- | --- | --- | --- | --- | --- | --- | --- | --- | --- | --- | --- | --- |
| Control(n=16)  ADR(n=17) | | | Control | ADR | Control | ADR | Control | ADR | Control | ADR | Control | ADR | Control | ADR | Control | ADR | Control | ADR | Control | ADR |
| **Baseline** | **After injection #2** | 1,000 | | 1 | <0,001 | <0,001 | <0,001 | <0,001 | <0,001 | <0,001 | 0,223 | 0,096 | <0,001 | <0,001 | 0,521 | <0,001 | 0,218 | 0,278 | 0,785 | 0,848 |
|  | **After injection #4** | 0,346 | | <0,001 | <0,001 | <0,001 | <0,001 | <0,001 | <0,001 | <0,001 | 1,000 | 1,000 | <0,001 | <0,001 | 0,142 | <0,001 | 0,749 | 0,848 | 0,179 | 0,076 |
|  | **ROSC 5 minutes** | <0,001 | | <0,001 | <0,001 | <0,001 | <0,001 | <0,001 | <0,001 | <0,001 | 0,025 | <0,001 | 0,634 | <0,001 | 0,194 | <0,001 | 0,179 | 0,012 | 0,005 | <0,001 |
|  | **ROSC 10 minutes** | <0,001 | | <0,001 | <0,001 | <0,001 | <0,001 | <0,001 | <0,001 | <0,001 | 0,723 | <0,001 | 1,000 | 1,000 | 0,248 | <0,001 | 0,073 | 0,149 | 0,008 | <0,001 |
| **After injection #2** | **Baseline** | 1,000 | | 1 | <0,001 | <0,001 | <0,001 | <0,001 | <0,001 | <0,001 | 0,223 | 0,096 | <0,001 | <0,001 | 0,521 | <0,001 | 0,218 | 0,278 | 0,785 | 0,848 |
|  | **After injection #4** | 1,000 | | <0,001 | 0,557 | <0,001 | 0,895 | <0,001 | 1,000 | 0,002 | 1,000 | 0,071 | 1,000 | 1,000 | 1,000 | 1,000 | 0,361 | 0,202 | 0,107 | 0,113 |
|  | **ROSC 5 minutes** | <,001 | | <0,001 | 0,251 | <0,001 | 0,075 | <0,001 | 0,095 | <0,001 | <0,001 | <0,001 | 0,158 | <,001 | 1,000 | 1,000 | 0,010 | <0,001 | 0,002 | <0,001 |
|  | **ROSC 10 minutes** | 0,002 | | <0,001 | 1,000 | <0,001 | 0,553 | <0,001 | 0,748 | <0,001 | <0,001 | <0,001 | 0,013 | <,001 | 1,000 | 1,000 | 0,003 | 0,012 | 0,004 | 0,001 |
| **After injection #4** | **Baseline** | 0,346 | | <0,001 | <0,001 | <0,001 | <0,001 | <0,001 | <0,001 | <0,001 | 1,000 | 1,000 | <0,001 | <0,001 | 0,142 | <0,001 | 0,749 | 0,848 | 0,179 | 0,076 |
|  | **After injection #2** | 1,000 | | <0,001 | 0,557 | <0,001 | 0,895 | <0,001 | 1,000 | 0,002 | 1,000 | 0,071 | 1,000 | 1,000 | 1,000 | 1,000 | 0,361 | 0,202 | 0,107 | 0,113 |
|  | **ROSC 5 minutes** | 0,002 | | <0,001 | 1,000 | 1,000 | 1,000 | 0,348 | 1,000 | 0,529 | <0,001 | <0,001 | 0,174 | <0,001 | 1,000 | 1,000 | 0,096 | 0,020 | 0,155 | 0,012 |
|  | **ROSC 10 minutes** | 0,184 | | <0,001 | 1,000 | 1,000 | 1,000 | 0,951 | 1,000 | 1,000 | 0,017 | <0,001 | 0,015 | <0,001 | 1,000 | 1,000 | 0,035 | 0,211 | 0,203 | 0,097 |
| **ROSC 5 minutes** | **Baseline** | <0,001 | | <0,001 | <0,001 | <0,001 | <0,001 | <0,001 | <0,001 | <0,001 | 0,025 | <0,001 | 0,634 | <0,001 | 0,194 | <0,001 | 0,179 | 0,012 | 0,005 | <0,001 |
|  | **After injection #2** | <0,001 | | <0,001 | 0,251 | <0,001 | 0,075 | <0,001 | 0,095 | <0,001 | <0,001 | <0,001 | 0,158 | <0,001 | 1,000 | 1,000 | 0,01 | <0,001 | 0,002 | <0,001 |
|  | **After injection #4** | 0,002 | | <0,001 | 1,000 | 1,000 | 1,000 | 0,348 | 1,000 | 0,529 | <0,001 | <0,001 | 0,174 | <0,001 | 1,000 | 1,000 | 0,096 | 0,020 | 0,155 | 0,012 |
|  | **ROSC 10 minutes** | 1,000 | | 1 | 1,000 | 1,000 | 1,000 | 1,000 | 1,000 | 1,000 | 1,000 | 0,694 | 1,000 | <0,001 | 1,000 | 1,000 | 0,637 | 0,286 | 0,897 | 0,398 |
| **ROSC 10 minutes** | **Baseline** | <0,001 | | <0,001 | <0,001 | <0,001 | <0,001 | <0,001 | <0,001 | <0,001 | 0,723 | <0,001 | 1,000 | 1,000 | 0,248 | <0,001 | 0,073 | 0,149 | 0,008 | <0,001 |
|  | **After injection #2** | 0,002 | | <0,001 | 1,000 | <0,001 | 0,553 | <0,001 | 0,748 | <0,001 | <0,001 | <0,001 | 0,013 | <0,001 | 1,000 | 1,000 | 0,003 | 0,012 | 0,004 | 0,001 |
|  | **After injection #4** | 0,184 | | <0,001 | 1,000 | 1,000 | 1,000 | 0,951 | 1,000 | 1,000 | 0,017 | <0,001 | 0,015 | <0,001 | 1,000 | 1,000 | 0,035 | 0,211 | 0,203 | 0,097 |
|  | **ROSC 5 minutes** | 1,000 | | 1 | 1,000 | 1,000 | 1,000 | 1,000 | 1,000 | 1,000 | 1,000 | 0,694 | 1,000 | <0,001 | 1,000 | 1,000 | 0,637 | 0,286 | 0,897 | 0,398 |
| BE = base excess, HCO_3_^-^ = hydrogen bicarbonate, ADR = adrenaline, pCO_2_ = partial pressure of carbon dioxide, pO_2_ = partial pressure of oxygen, sO_2_ = saturation Oxygen, P-values for pH, BE, HCO_3_^-^, lactate, haemoglobin, potassium, pCO_2_, are calculated with a variance test and Bonferroni correction. While for pO_2_ and sO_2_, the Kruskal-Wallis test and Bonferroni correction were used. Significance was set at p<0.05. | | | | | | | | | | | | | | | | | | | | |

**Supplement Table 2**. **Comparisons of differences within the control non-ROSC group and the ADR non-ROSC group**

|  |  | | **pH** | | **BE** | | **HCO3^-^** | | **Lactate** | | **pCO_2_** | | **Potassium** | | **Haemoglobin** | | **pO_2_** | | **sO_2_** | |  |
| --- | --- | --- | --- | --- | --- | --- | --- | --- | --- | --- | --- | --- | --- | --- | --- | --- | --- | --- | --- | --- | --- |
| **Control(n=8)**  **ADR(=19)** |  | **Control** | | **ADR** | **Control** | **ADR** | **Control** | **ADR** | **Control** | **ADR** | **Control** | **ADR** | **Control** | **ADR** | **Control** | **ADR** | **Control** | **ADR** | **Control** | **ADR** |  |
| **Baseline** | **After injection #2** | | 0.010 | 1.00 | 0.189 | <0.001 | <0.001 | <0.001 | 0.004 | <0.001 | 1.000 | 0.009 | 0.040 | <0.001 | 0.065 | <0.001 | 0.576 | <0.001 | 0.983 | 0.490 |  |
|  | **After injection #4** | | <0.001 | <0.001 | 0.310 | <0.001 | <0.001 | <0.001 | 0.001 | <0.001 | 1.000 | 0.313 | 0.040 | <0.001 | 0.112 | <0.001 | 0.389 | 0.004 | 0.590 | 0.044 |  |
|  |  | |  |  |  |  |  |  |  |  |  |  |  |  |  |  |  |  |  |  |  |
| **After injection #2** | **Baseline** | | 0.010 | 1.000 | 0.189 | <0.001 | <0.001 | <0.001 | 0.004 | <0.001 | 1.000 | 0.009 | 0.040 | <0.001 | 0.065 | <0.001 | 0.576 | <0.001 | 0.983 | 0.490 |  |
|  | **After injection #4** | | 0.885 | 0.001 | 1.000 | <0.001 | 0.272 | <0.001 | 1.000 | <0.001 | 1.000 | 0.446 | 1.000 | 0.047 | 1.000 | 1.000 | 0.762 | 0.395 | 0,575 | 0.187 |  |
|  |  | |  |  |  |  |  |  |  |  |  |  |  |  |  |  |  |  |  |  |  |
| **After injection #4** | **Baseline** | | <0.001 | <0.001 | 0.310 | <0.001 | <0.001 | <0.001 | 0.001 | <0.001 | 1.000 | 0.313 | 0.040 | <0.001 | 0.112 | <0.001 | 0.389 | 0.004 | 0.590 | 0.044 |  |
|  | **After injection #2** | | 0.885 | 0.001 | 1.000 | <0.001 | 0.272 | <0.001 | 1.000 | <0.001 | 1.000 | 0.446 | 1.000 | 0.047 | 1.000 | 1.000 | 0.762 | 0.395 | 0,575 | 0.187 |  |
| BE = base excess, HCO_3_^-^ = hydrogen bicarbonate, ADR = adrenaline, pCO_2_ = partial pressure of carbon dioxide, pO_2_ = partial pressure of oxygen, sO_2_ = saturation Oxygen, P-values for pH, BE, HCO_3_^-^, lactate, haemoglobin, potassium, pCO_2_, are calculated with a variance test and Bonferroni correction. While for pO_2_ and sO_2_, the Kruskal-Wallis test and Bonferroni correction were used. Significance was set at p<0.05. | | | | | | | | | | | | | | | | | | | | | |

**Supplement Table 3**. **Comparisons of differences within the total control group and the total ADR group**

|  |  | **pH** | | **BE** | | **HCO_3_^-^** | | **Lactate** | | **pCO_2_** | | **Potassium** | | **Haemoglobin** | | **pO_2_** | | **sO_2_** | |
| --- | --- | --- | --- | --- | --- | --- | --- | --- | --- | --- | --- | --- | --- | --- | --- | --- | --- | --- | --- |
| **Control(n=24)**  **ADR(n=36)** |  | **Control** | **ADR** | **Control** | **ADR** | **Control** | **ADR** | **Control** | **ADR** | **Control** | **ADR** | **Control** | **ADR** | **Control** | **ADR** | **Control** | **ADR** | **Control** | **ADR** |
| **Baseline** | **After injection #2** | 0.299 | 1.000 | <0.001 | <0.001 | <0.001 | <0.001 | <0.001 | <0.001 | 0.447 | <0.001 | <0.001 | <0.001 | 0.010 | <0.001 | 0.098 | <0.001 | 0.636 | 0.304 |
|  | **After injection #4** | 0.005 | <0.001 | <0.001 | <0.001 | <0.001 | <0.001 | <0.001 | <0.001 | 1.000 | 0.978 | <0.001 | <0.001 | 0.005 | <0.001 | 0.290 | 0.020 | 0.509 | 0.557 |
|  |  |  |  |  |  |  |  |  |  |  |  |  |  |  |  |  |  |  |  |
| **After injection #2** | **Baseline** | 0.299 | 1.000 | <0.001 | <0.001 | <0.001 | <0.001 | <0.001 | <0.001 | 0.447 | <0.001 | <0.001 | <0.001 | 0.010 | <0.001 | 0.098 | <0.001 | 0.636 | 0.304 |
|  | **After injection #4** | 0.340 | <0.001 | 0.927 | <0.001 | 0.049 | <0.001 | 0.230 | <0.001 | 1.000 | 0.005 | 1.000 | 0.193 | 1.000 | 1.000 | 0.552 | 0.184 | 0.259 | 0.106 |
|  |  |  |  |  |  |  |  |  |  |  |  |  |  |  |  |  |  |  |  |
| **After injection #4** | **Baseline** | 0.005 | <0.001 | <0.001 | <0.001 | <0.001 | <0.001 | <0.001 | <0.001 | 1.000 | 0.978 | <0.001 | <0.001 | 0.005 | <0.001 | 0.290 | 0.020 | 0.509 | 0.557 |
|  | **After injection #2** | 0.340 | <0.001 | 0.927 | <0.001 | 0.049 | <0.001 | 0.230 | <0.001 | 1.000 | 0.005 | 1.000 | 0.193 | 1.000 | 1.000 | 0.552 | 0.184 | 0.259 | 0.106 |

BE = base excess, HCO_3_^-^ = hydrogen bicarbonate, ADR = adrenaline, pCO_2_ = partial pressure of carbon dioxide, pO_2_ = partial pressure of oxygen, sO_2_ = saturation of oxygen. P-values for BE, HCO_3_^-^, lactate, pCO_2_, potassium, haemoglobin, are calculated with a variance test and Bonferroni correction, while for pO_2_ and sO_2_, the Kruskal-Wallis test and Bonferroni correction were used. Significance was set at p<0.05.
